# Molecular mechanisms of retroviral integration site selection

**DOI:** 10.1093/nar/gku769

**Published:** 2014-08-21

**Authors:** Mamuka Kvaratskhelia, Amit Sharma, Ross C. Larue, Erik Serrao, Alan Engelman

**Affiliations:** 1 Center for Retrovirus Research and College of Pharmacy, The Ohio State University, Columbus, OH 43210, USA; 2 Department of Cancer Immunology and AIDS, Dana-Farber Cancer Institute and Department of Medicine, Harvard Medical School, Boston, MA 02215, USA

## Abstract

Retroviral replication proceeds through an obligate integrated DNA provirus, making retroviral vectors attractive vehicles for human gene-therapy. Though most of the host cell genome is available for integration, the process of integration site selection is not random. Retroviruses differ in their choice of chromatin-associated features and also prefer particular nucleotide sequences at the point of insertion. Lentiviruses including HIV-1 preferentially integrate within the bodies of active genes, whereas the prototypical gammaretrovirus Moloney murine leukemia virus (MoMLV) favors strong enhancers and active gene promoter regions. Integration is catalyzed by the viral integrase protein, and recent research has demonstrated that HIV-1 and MoMLV targeting preferences are in large part guided by integrase-interacting host factors (LEDGF/p75 for HIV-1 and BET proteins for MoMLV) that tether viral intasomes to chromatin. In each case, the selectivity of epigenetic marks on histones recognized by the protein tether helps to determine the integration distribution. In contrast, nucleotide preferences at integration sites seem to be governed by the ability for the integrase protein to locally bend the DNA duplex for pairwise insertion of the viral DNA ends. We discuss approaches to alter integration site selection that could potentially improve the safety of retroviral vectors in the clinic.

## INTRODUCTION

Retroviral replication requires the covalent integration of the reverse transcribed viral genome into the host cell chromatin. The integrated form of the virus, referred to as the provirus, provides a template for viral gene expression. Because the provirus is an integral part of the host genome, retroviruses persist in the host for the lifetime of the infected cell. This trait of irreversible integration makes retroviruses particularly attractive vehicles for human-based genetic therapy (1).

Although most of the host cell genome is amenable to integration (2), retroviral integration is not a random process (3), with several factors influencing integration site selectivity. There are seven different retroviral genera—alpha through epsilon, lenti and spuma—and the selection of host DNA sequence and chromatin-associated features seems to largely follow genera-specific patterns (4,5). For examples, lentiviruses including HIV-1 prefer to integrate within the bodies of active genes located within gene dense regions of chromosomes (6), while gammaretroviruses such as Moloney murine leukemia virus (MoMLV) display bias for integrating in the vicinity of strong enhancers, active gene promoters and associated CpG islands (7–9). The deltaretrovirus human T-lymphotropic virus type 1 and the alpharetrovirus avian sarcoma-leukosis virus (ASLV) each display a pattern that differs from HIV-1 and MoMLV, as neither shows a strong preference for active genes or transcription start sites (TSSs) (4,10). The betaretrovirus mouse mammary tumor virus (MMTV) seems the least selective of all, displaying an integration pattern on the genomic level that is basically indistinguishable from random (11,12).

Studies of the mechanisms of retroviral integration have revealed two key players that determine integration site selection: the retroviral integrase (IN) protein and cognate cellular binding partners (13,14). In the case of lentiviral INs, integration site targeting is in large part guided by the cellular chromatin binding protein lens epithelium-derived growth factor (LEDGF)/p75, which facilitates integration into active gene bodies (15–18). More recent studies have identified the bromo- and extra-terminal domain (BET) proteins (bromodomain (BRD) proteins 2, 3 and 4) as the main cellular binding partners of MoMLV IN and demonstrated their role in promoting efficient MoMLV integration near TSSs (19–21). Collectively, these findings have provided clues as to why different retroviruses exhibit markedly distinct integration site selectivity. Although retroviruses from the other five genera show less dramatic targeting of chromatin-associated features than do either the lentiviruses or gammaretroviruses, we nonetheless expect that these IN proteins also interact with specific nuclear factors to facilitate virus integration.

The significance of integration site selection has been highlighted by studies that have used retroviral vectors in human gene-therapy. Retroviruses present efficient vehicles for the delivery of therapeutic genes due to their trait of stable DNA integration and because they are amenable to pseudotyping with a variety of envelope glycoproteins (1,22,23). In particular, MoMLV-based vectors have been successfully utilized in the treatment of primary immunodeficiencies (24,25). However, adverse effects associated with integration of MoMLV-based vectors near proto-oncogenes were observed in these clinical trials (25–28). Therefore, understanding the underlying mechanisms for integration site specificity could lead to the development of safer vectors for human gene-therapy. The recent identification of BET proteins as principal binding partners of MoMLV IN offers a new means to understand and address this problem. The present review compares the mechanisms of action of LEDGF/p75 and BET proteins in their ability to navigate HIV-1 and MoMLV integration to select chromatin sites and the implications for human gene-therapy.

## INTEGRATION: CATALYTIC MECHANISM AND TARGET SITE SELECTION

Retroviral IN exhibits two distinct catalytic activities, termed 3′ processing and strand transfer, to covalently insert the viral DNA into the host genome. Productive 3′ processing involves the cleavage of a dinucleotide from the 3′ ends of the viral DNA, yielding invariant CA_OH_-3′ sequences. During the following strand transfer reaction, IN uses these 3′-hydroxyl groups to generate a staggered cut in complementary strands of the target DNA and concomitantly join the viral DNA ends to the host genome (29,30). The different retroviral IN proteins recognize scissile phosphodiester bonds in target DNA that are separated by 4–6 bp for strand transfer. The single-strand gaps in the DNA recombination intermediate are repaired by cellular enzymes, which accordingly yield 4–6 bp duplications of target DNA flanking the integrated provirus. In infected cells, IN functions in the context of a large nucleoprotein complex called the preintegration complex (PIC), which in addition to IN and viral DNA is comprised of a number of viral and cellular proteins.

X-ray crystal structures of the spumaviral prototype foamy virus (PFV) IN in complex with viral and target DNA substrates have provided a major breakthrough for understanding the mechanism of integration (31,32). One key feature observed in the functional complex is that the target DNA is significantly kinked to optimally position IN active sites for the pair-wise strand transfer events. These findings augmented earlier biochemical data (33–37) showing that IN favors integration into DNA acceptor sites that display inherent bendability, including nucleosomal DNA wrapped around core histones. In particular, the widened major groove, where nucleosomal DNA is relatively distorted, appears to be preferentially targeted by retroviral INs. Retroviral INs additionally exhibit a preference for weakly conserved palindromic sequences that center around the staggered cut in target DNA (3,38–41). It is logical that these sequences are only weakly conserved, as strong nucleotide sequence specificity would be disadvantageous for viral fitness since this would limit the distribution of proviral sites suitable for optimal gene expression. Accordingly, the majority of contacts between PFV IN and target DNA in the crystal structures are mediated through the phosphodiester backbone (31).

Recent research has indicated that the nature of the palindromic sequence in large part underlies the bendability of the substrate at sites of viral DNA joining. In particular, the preferred PFV integration site (-3)KWK↓VYRBMWM(6) (written using International Union of Biochemistry base codes; the arrow marks the position of viral DNA plus-strand insertion and the underline notes the target site duplication, which is 4 bp for PFV) (42) preferentially harbors the YR dinucleotide at the center of the integration site. The varying combinations of purine (R)/pyrimidine (Y) dinucleotides possess inherently different base stacking propensity and hence flexibility: YR and RY are the most and least distortable, respectively, while RR and YY fall in between (43). Retroviral IN proteins harbor three common domains: the zinc-chelating N-terminal domain (NTD), central catalytic core domain (CCD) that harbors the enzyme active site and C-terminal domain (CTD) (reviewed in 44). Because PFV IN amino acids Ala188 in the CCD and Arg329 in the CTD interact with the eight bases of the target DNA consensus nucleotide signature that abut the central YR, the DNA palindrome represents preferentially bendable sequences that result from PFV IN-base interactions and the centrally flexible YR sequence (31). Similar scenarios are likely for the other retroviruses. Recent analysis of the consensus HIV-1 integration site (-3)TDG↓(G/V)TWA(C/B)CHA(7) highlighted the dinucleotide signature (0)RYXRY(4) at its center. Though enriched in rigid RY dinucleotides at first glance, this pattern actually ensures for relatively flexible sequences overlapping the center of the presumed DNA bend: Y at the center X yields YY and YR at nucleotide positions 1 and 2 and at 2 and 3, respectively, whereas R in the center yields YR and RR at these respective positions. Due to the lack of HIV-1 IN-DNA structures, less is known about the details of IN-target DNA contacts that abut these central flexible motifs than is known for PFV IN. Nevertheless, mutagenesis studies indicate that HIV-1 IN CCD residue Ser119, like its Ala188 analog in PFV IN, interacts with the bases that lie three positions upstream from the points of viral DNA joining (45).

## LEDGF/p75 AND HIV-1 INTEGRATION

LEDGF/p75 was identified as an HIV-1 IN binding protein using different proteomic screens, including mass spectrometry (MS)-based analysis of cellular factors associated with ectopically expressed IN in human cells and by yeast 2-hybrid (46–49). Because LEDGF/p75 knockdown significantly reduced the steady-state level of ectopically expressed HIV-1 IN, the host factor appeared to be the principal cellular binding partner of the viral protein in human cells (50,51).

LEDGF/p75 is a ubiquitous cellular protein. It was initially discovered as a human transcriptional coactivator (52) and has been shown to move around the nucleus of living cells by interacting with chromatin in a hop/scan mode that is common among transcription factors (53). LEDGF/p75 also interacts with a number of cellular proteins including JPO2 (54,55), Cdc7-activator of S-phase kinase (ASK) (56), the ‘domesticated’ transposase pogZ (57) and menin, which links LEDGF/p75 with mixed-lineage leukemia (MLL) histone methyltransferase and results in MLL-dependent transcription and leukemic transformation (58).

**Figure 1.**
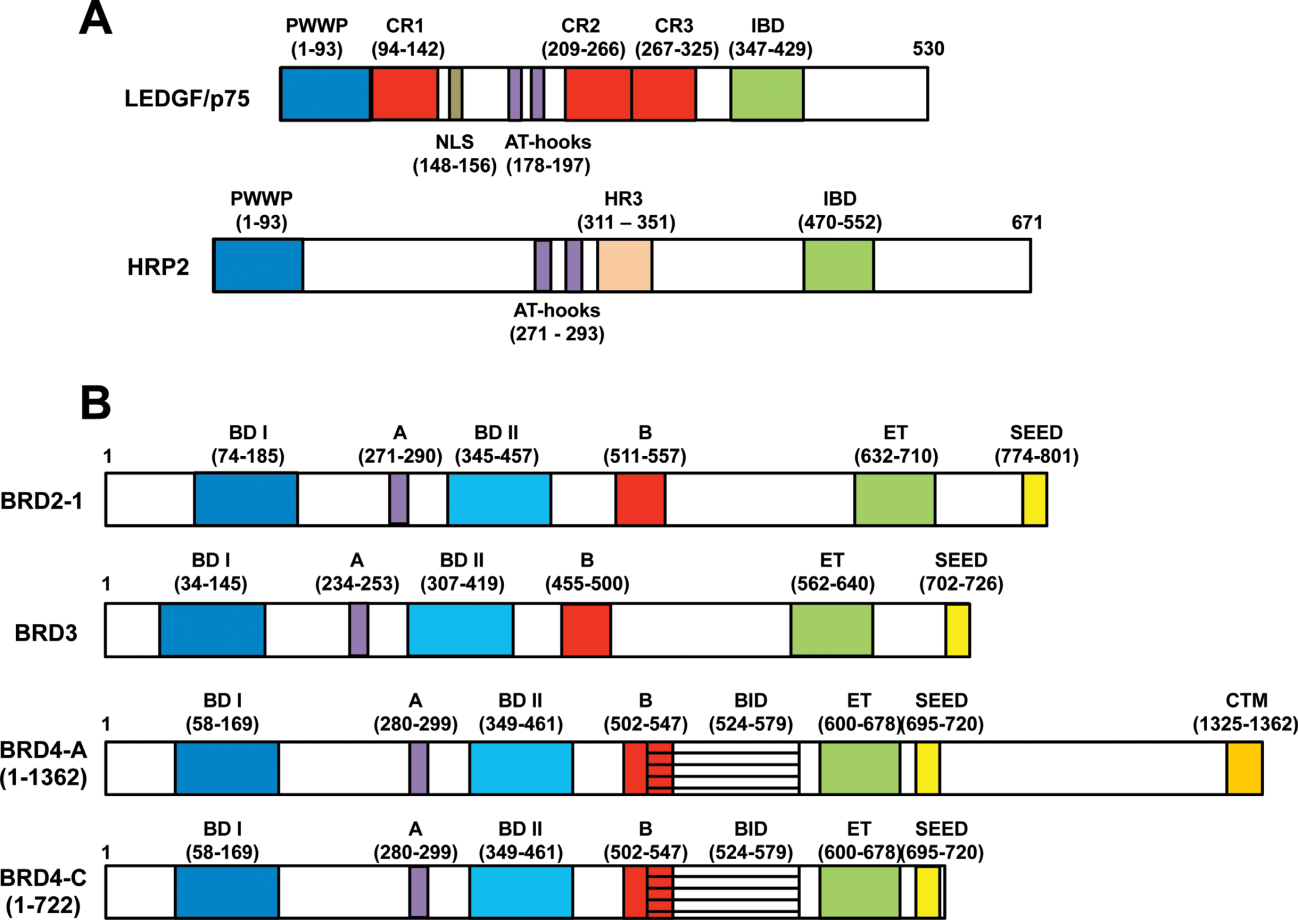
Features and domain organization of LEDGF/p75, HRP2 and BET proteins. (**A**) The N-terminal region of LEDGF/p75, which contains a PWWP domain, charged regions (CR) 1–3, nuclear localization signal (NLS), and AT-hooks, interacts with chromatin. Similar to LEDGF/p75, HRP2 contains an N-terminal PWWP domain and AT-hooks. HRP2 has an additional domain, termed the homology region III (HR3) that is conserved in multiple HRP2 homologs as well as in LEDGF/p75. The C-terminal regions of both proteins exhibit the IBD that directly interacts with lentiviral INs. (**B**) The BET proteins consist of BRD2, 3, 4 and T (not pictured). Whereas BRD3 is expressed as a single isoform, BRD2 is expressed as four isoforms (isoform 1 is pictured) and BRD4 as three isoforms (isoforms A and C are pictured; as compared to isoform C, isoform B harbors a unique 75 amino acid C-terminal tail that interacts with condensing II complexes; 183). Known domains and their respective start and end amino acids numbers are indicated. Two N-terminal bromodomains (BD I and II) and motifs A and B collectively contribute to high affinity chromatin binding. In the C-terminal region of the BET proteins, the conserved ET domain interacts with multiple proteins including MoMLV IN. Other domains include the SEED domain, which is present in all BET proteins, the BID, which is present in all BRD4 isoforms, and the CTM, which is unique to BRD4 isoform A.

The structural organization of LEDGF/p75 (Figure 1A) reveals an N-terminal PWWP domain, a basic-type nuclear localization signal, two AT-hook DNA binding motifs and three highly charged regions (CR1–3) that allow this protein to tightly engage chromatin throughout the cell cycle (59,60). The C-terminal region contains a domain that is termed the IN binding domain (IBD) for its ability to directly interact with HIV-1 IN (61). LEDGF/p75 belongs to the hepatoma-derived growth factor related protein (HRP) family that comprises five additional members (HDGF, HRP1–3 and LEDGF/p52). HRP2 is the only other known cellular protein that contains both an IBD and PWWP domain (Figure 1A). The other family members, including the smaller alternatively spliced isoform of LEDGF (LEDGF/p52), lack the IBD and thus fail to interact with HIV-1 IN (50).

LEDGF/p75 binds tightly to a number of lentiviral INs but fails to interact with INs from the other retroviral genera (62–64). Accordingly, *in vitro* assays with purified INs have revealed that LEDGF/p75 significantly stimulated the strand transfer activities of lentiviral but not of other retroviral INs (47,60,61,64). Initially it was unclear as to whether LEDGF/p75 also promoted efficient HIV-1 integration in cells, as significant knockdown of LEDGF/p75 either failed to reduce infectious HIV-1 titer (62) or yielded only ∼2-fold reductions in integration (65,66). However, parallel findings indicated that residual amounts of chromatin-associated LEDGF/p75, which could persist among cell clones despite an overall efficient level of knockdown, were sufficient to support near wild-type levels of HIV-1 infection and integration (67). Consistent with this finding, studies using LEDGF/p75 knockout cells revealed 5 to 80-fold defects in HIV-1 titer associated with ∼2 to 12-fold reductions in integration (16–18,68). The integration defect of the ungulate lentivirus equine infectious anemia virus (EIAV) in mouse knockout cells was reportedly >50-fold (16). Significant inhibitory effects on HIV-1 replication were also observed in cells engineered to express constructs that contained the IBD but lacked the N-terminal elements present in full length LEDGF/p75 that confer chromatin binding (67,69,70). The monitoring of viral replication intermediates has pinpointed that LEDGF/p75 depletion or overexpression of dominant-interfering IBD constructs does not significantly affect HIV-1 reverse transcription but instead selectively impairs integration. Collectively, the *in vitro* and cell culture experiments conclusively demonstrated a stimulatory role for LEDGF/p75 on lentiviral DNA integration.

Genome-wide integration site mapping experiments were carried out to explore the role of LEDGF/p75 in integration site selectivity. The first line of evidence for its role in HIV-1 target site selection emerged from the analysis of LEDGF/p75 knockdown cells, where significantly reduced frequencies of HIV-1 integration into active genes were observed even though these cells supported normal levels of HIV-1 infection (15,62). Subsequent experiments using LEDGF/p75 knockout cells corroborated these findings and extended them to show that a significant percentage of HIV-1 proviruses were aberrantly located near TSSs in the absence of the host factor (16,17). Furthermore, chimeric constructs that replaced the N-terminal chromatin-binding portions of LEDGF/p75 with the chromatin binding regions of other proteins supported efficient HIV-1 infection (71–73) and retargeted integration away from active genes and toward the sites preferentially bound by the heterologous chromatin binding domains (72–74). For example, replacing the N-terminal PWWP domain and AT hooks of LEDGF/p75 with a plant homeodomain (PHD) finger redirected HIV-1 DNA integration to TSSs. Integration frequencies within 2.5 kb of TSSs were 50.3 and 3.8% in the presence of the ectopically expressed fusion and wild-type LEDGF/p75 proteins, respectively (72). Similar use of the chromobox homolog 1 (CBX1) and heterochromatin protein 1 (HP1) alpha chromatin binding modules imposed a genomic pattern of HIV-1 integration that resembled random (72,73).

Mapping of the LEDGF/p75 chromatin-binding profile along the Encyclopedia of DNA Elements has revealed a preference for binding active transcription units, which paralleled the enhanced HIV-1 integration frequencies at these locations (75). These observations extended the prior finding that LEDGF/p75 was required for the chromosomal association of ectopically expressed HIV-1 IN in human cells (50). Collectively, these findings provide strong evidence that LEDGF/p75 tethers PICs to active transcription units during HIV-1 integration. Although LEDGF/p75 can potently stimulate HIV-1 IN catalytic function *in vitro* (47,60,61,76,77), it is somewhat unclear if the host factor provides this function during virus infection. LEDGF/p75 immunoprecipitation can recover PIC activity from infected cell extracts, indicating that the IN-binding factor is a component of the HIV-1 PIC (62). Interestingly, the wild-type level of HIV-1 PIC activity is maintained in samples isolated from LEDGF/p75 knockout cells (17). Therefore, LEDGF/p75 may provide chromatin-tethering function to lentiviral PICs without contributing to the formation of the catalytically active intasome.

The frequency of HIV-1 integration into active transcription units remained greater than random in LEDGF/p75 knockout cells (16–18), suggesting a potential role for other cellular proteins in integration targeting. In particular, a role for HRP2 was investigated due to its close structural similarity with LEDGF/p75 (Figure 1A). *In vitro* assays with purified proteins demonstrated that HRP2 tightly binds HIV-1 IN and significantly stimulates its catalytic function (61). However, unlike LEDGF/p75, HRP2 does not remain bound to chromatin throughout the cell cycle (78). HRP2 depletion in cells containing normal levels of LEDGF/p75 did not have any detectable effect on HIV-1 titer or integration targeting (18,67,79,80). When HRP2 was depleted in LEDGF/p75 knockout cells, a further reduction in the preference of HIV-1 for integrating into active genes was observed (80,81). These findings argue that LEDGF/p75 is the principal cellular determinant for targeting HIV-1 integration to active transcriptional units and that HRP2 could play a secondary role. Notably, the preference for HIV-1 to integrate into active genes remained greater than random in LEDGF/HRP2 double knockout cells, suggesting that subsidiary targeting roles might exist for as of yet undefined lentiviral IN-binding proteins (80).

## BET PROTEINS AND MoMLV INTEGRATION

The observation that the distribution of MoMLV proviruses along chromatin differed markedly from HIV-1 suggested that MoMLV IN relies on cellular binding partners other than LEDGF/p75 for integration target-site selection. Initial experiments with yeast 2-hybrid screening revealed a number of potential binding partners of MoMLV IN, including BRD2 (82). More recent MS-based proteomic analysis of human cellular proteins that co-purified with recombinantly expressed MoMLV IN identified BET proteins (BRD2, 3 and 4) as main binding partners of the viral protein (19,21).

BRD2, 3, 4 and T belong to the BET protein family, which in turn is a part of the extended BET family that includes BRD1, 7, 8 and 9. BRD2, 3 and 4 are ubiquitously expressed, whereas BRDT is only expressed in testis. BET proteins have been implicated in transcription, DNA replication and cell cycle control (reviewed in 83,84). They exhibit several conserved domains and motifs (Figure 1B). Two N-terminal bromodomains (BD-I and BD-II) bind acetylated H3 and H4 tails on chromatin (85,86). Two conserved motifs, A and B, are positively charged and could contribute to DNA binding (87). An additional basic residue-enriched interaction domain (BID) has recently been described for BRD4 and shown to interact with cellular factor p53 (88). While this domain has not been confirmed in BRD2 or BRD3, sequence alignments identify a similar region corresponding to BRD2 residues 533–584 that is 44% identical and 66% homologous considering conservative amino acid substitutions. A short 17-residue region of BRD3 (amino acids 476–493) shows homology to the N-terminal part of the BID, though this could be due to the overlapping B motif. The C-terminal extra-terminal (ET) and SEED (Ser/Glu/Asp-rich region) domains that are present throughout the BET proteins directly engage various cellular proteins including transcription factors, chromatin modifying proteins, histone modifying enzymes and also interact with different viral proteins (reviewed in 84). BRD3 exhibits a single isoform, whereas BRD2 and BRD4 are expressed in several isoforms (Figure 1B). In addition to the above structural elements, BRD4 isoform C contains a C-terminal motif (CTM) that has been shown to bind a number of cellular proteins and also plays a role in HIV-1 latency (89–91).

Notably, BET proteins play an active role in the life cycles of different viral families including Papillomaviridae, Herpesviridae, Polyomaviridae and Retroviridae. In addition to regulating transcriptional activation of Epstein–Barr virus, Kaposi's sarcoma-associated herpesvirus (KSHV), and papillomavirus, they can repress the transcription of papillomavirus E6 and E7 promoters, aid papillomavirus episomal maintenance and genome segregation, and control reactivation of latent HIV-1 reservoirs (reviewed in 83,92; also see 90,91,93–96). Of these, the most pertinent to this review is the role of BET proteins in tethering papillomavirus genomes to condensed mitotic chromosomes (93,94), which is mediated through the binding of BRD4 with papillomavirus E2 protein (93,97,98). In particular, the C-terminal DNA-binding domain of E2 protein binds viral DNA, whereas the N-terminal transactivation domain of the E2 protein directly interacts with the C-terminal region of BRD4. In turn, this nucleoprotein complex is tethered to host chromatin by the two N-terminal bromodomains of BRD4, which associates with acetylated lysines in the tail regions of histones H3 and H4 (83,86,99,100). This tethering mechanism ensures papillomavirus episomal maintenance by coupling the viral genomes to host chromosomes during mitosis, and subsequent distribution to daughter cells after cell division.

Investigation into the role of BET proteins during MoMLV integration has also revealed a bimodal tethering mechanism. Biochemical assays with purified proteins have revealed direct, high affinity interactions between BET proteins and MoMLV IN as well as between BET proteins and mononucleosomes (19–21,87). Furthermore, purified recombinant BRD4-C (19,87) and, to a lesser degree, the isolated ET domains of BRD2, 3 and 4 (20), significantly enhanced the pair-wise or concerted integration activity of MoMLV IN *in vitro* (19,20,87). BET protein binding and enzymatic stimulation was specific for IN proteins derived from gammaretroviruses and not for other retroviruses (19–21). The stimulation of MoMLV IN *in vitro* activity was mediated through the bimodal interaction of BRD4 with naked DNA and MoMLV IN (87). Interestingly, the levels of stimulation of MoMLV IN integration activities by BRD4 were comparable to that of HIV-1 IN by LEDGF/p75 (19). In addition, MoMLV IN and BET proteins have been shown to colocalize in cell nuclei (20,21).

Small molecules JQ-1 and I-BET, which specifically impair interactions of all three BET proteins with cognate histone marks (86,101), were exploited to examine the role of BET proteins in MoMLV replication. These inhibitors selectively impaired MoMLV but not HIV-1 replication (19–21). Furthermore, the analysis of replication intermediates revealed that the inhibition of BET proteins with JQ-1 impaired MoMLV integration in a dose dependent manner, yielding inhibitory concentration 50% (IC_50_) values of ∼50–100 nM (19,20). Taken together, *in vitro* and cell culture experiments indicate that the BET proteins function for MoMLV like LEDGF/p75 does for HIV-1: specific chromatin tethers that interact with PICs by binding their cognate IN and potentially stimulating its enzymatic function.

The chromatin binding sites of BET proteins have been mapped using ChIP-Seq experiments (100), which when compared with retroviral integration sites showed a positive correlation with MoMLV but not with HIV-1 or ASLV (19,21). In particular, MoMLV exhibited a strong preference for promoters associated with the binding sites of BET proteins. Treatment with JQ-1 and I-BET was used to experimentally examine the roles of BET proteins in MoMLV integration site selectivity (19,20). Alternatively, the effect of concurrent down-regulation of BRD2, 3 and 4 by a pool of short interfering (si) RNAs was investigated (19). Treatment with inhibitors or siRNA significantly reduced the characteristic preference of MoMLV for integrating near TSSs. For example, in the absence of JQ-1, MoMLV integration in HEK293T cells was strongly favored (39% integration events) within 2-kb regions of RefSeq TSSs, whereas after JQ-1 treatment this frequency was reduced in a dose-dependent manner to 11% at the highest dose tested (19). A complementary approach investigated an artificial fusion protein that contained the N-terminal chromatin binding segment of LEDGF/p75 (amino acids 1–324) and the C-terminal BRD4(ET/SEED) segment that interacts with MoMLV IN (21). Ectopic expression of the chimeric LEDGF(1–324)/BRD4(ET/SEED) protein retargeted MoMLV integration away from TSSs and toward the bodies of active genes, a pattern that is reminiscent of LEDGF/p75-mediated lentiviral integration. Taken together, these studies (19–21) have dissected the role of BET proteins in targeting MoMLV integration near TSSs.

## STRUCTURAL ASPECTS OF HIV-1 IN-LEDGF/p75 AND MoMLV IN-BET PROTEIN INTERACTIONS

**Figure 2.**
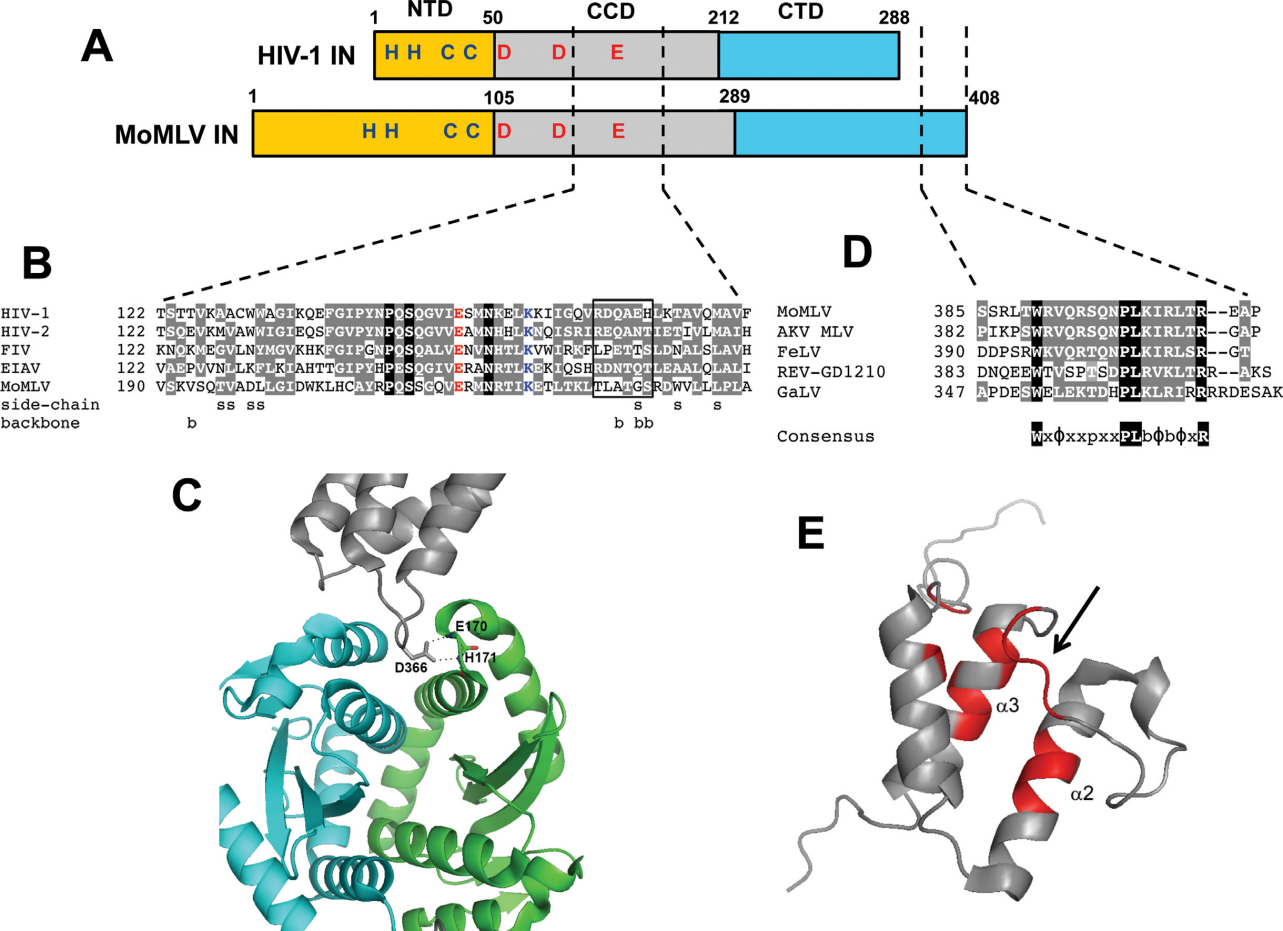
HIV-1 and MoMLV IN similarities and differences. (**A**) Features and domain organization of HIV-1 and MoMLV INs. Retroviral INs consist of three conserved domains, the N-terminal domain (NTD, yellow), the catalytic core domain (CCD, gray) and the C-terminal domain (CTD, blue). Shown in red is the conserved amino acids of the catalytic triad (DDE) that coordinates Mg^2+^ and is responsible for 3′ processing and strand transfer activities. Also shown in blue letters is the Zn binding motif (HH-CC type) that helps to mediate IN multimerization. (**B**) Sequence alignment of mid region sections of retroviral IN CCDs from HIV-1 strain NL4–3 (GenBank accession code M19921.2), HIV-2 strain ROD (M15390), feline immunodeficiency virus (FIV, M25381.1), equine infectious leukemia virus (EIAV, M16575.1) and MoMLV (NC_001501.1). Invariant residues across retroviral INs are shown in red (glutamic acid of the DDE catalytic triad) and blue (lysine that mediates binding to viral DNA; 103,184). Residues highlighted in black are identical across this alignment, whereas those highlighted in gray are conserved in minimally three of the sequences based on the following chemical groupings: G, A, S, T, P; M, V, L, I; F, Y, W; D, E, N, Q; K, R, H; C (185). IN residues that interact with the LEDGF/p75 IBD are highlighted by the nature of the contact: s for side chain and b for backbone (64). The rectangle highlights residues that compose the α4/5 connector region that lies between CCD α helices 4 and 5 and mediates several key contacts with LEDGF/p75 (108). (**C**) Ribbon diagram of the crystal structure of a dimer of the HIV-1 IN CCD (cyan and green) bound to the LEDGF/p75 IBD (gray). The carboxylate side chain of LEDGF/p75 residue Asp366 hydrogen bonds with the backbone amides of IN residues Glu170 and His171. The adjacent LEDGF/p75 residue Ile365 (not shown) predominantly interacts with the cyan IN molecule through hydrophobic contacts. (**D**) Sequence alignment of the C-terminal tail regions of gammaretroviral INs from the following full-length molecular clones: MoMLV, MLV from the AKV mouse strain (J01998.1), feline leukemia virus (FeLV, NC_001940.1), gibbon ape leukemia virus (GaLV, NC_001885.2) and reticuloendotheliosis virus (REV) strain GD1210 (KF709431.1). Among these viruses, MoMLV and FeLV have been shown to favor TSSs during integration (7,186). These IN proteins have also been shown to bind BET proteins *in vitro* (19–21). Below the alignment is the consensus WxΦxxpxxPLbΦbΦxR sequence, where p stands for small polar (S or T) residue, b stands for basic (R, K, or H), Φ stands for small hydrophobic (M, V, I, or L) and x refers to a position that is not conserved across the alignment. (**E**) Ribbon diagram of the NMR structure of BRD4 ET domain, with residues in red implicated in interacting with the MoMLV IN CTD as determined by chemical shift perturbations. These interactions were predominantly observed in helices 2 and 3 and the short loop connecting them (indicated by an arrow).

Highly conserved structural features of retroviral IN proteins include the catalytic triad of Asp, Asp and Glu (DDE) residues that coordinates a pair of essential Mg^2+^ ions during 3′ processing and strand transfer (32) and the Zn-binding motif (HH-CC type) in the NTD that contributes to IN multimerization and DNA binding (102,103) (Figure 2A). Furthermore, the crystal structure of the PFV IN-viral DNA complex, or intasome (103), has enabled plausible molecular modeling studies of HIV-1 IN interactions with its DNA substrates (45,104–107). These studies in turn suggest that the overall architectures of different retroviral intasomes may exhibit significant resemblance with the PFV structure. However, despite potential overall similarity among different retroviral intasome structures, studies with LEDGF/p75 and BET proteins have revealed that retroviruses from different genera markedly differ in their interactions with their cognate cellular binding partners.

The principal LEDGF/p75 IBD binding determinant on HIV-1 IN is the CCD (108), although the NTD is additionally required for high affinity binding (50,77,109). A short interhelical loop from the IBD docks into a narrow, V-shaped cavity at the interface of two IN CCD molecules and establishes functionally critical hydrogen bonds between LEDGF/p75 hotspot residue Asp366 and the backbone amides of IN residues Glu170 and His171 (Figure 2B and C) (108,110). The LEDGF/p75 binding pocket is conserved amongst lentiviral INs, whereas the corresponding segments in other retroviral INs exhibit significant differences (108). While the dimeric organization of the PFV CCDs is present in the functional intasome, the two interacting subunits of the PFV CCD create an ∼90° angle compared with an acute angle observed for the lentiviral CCDs. Additionally, IN CCD residues that interact with LEDGF/p75 show greater degrees of conservation amongst lentiviral as compared to the other retroviral proteins despite the fact that all retroviral IN CCDs contain the invariant DDE catalytic triad (64,109,111; also see Figure 2B).

Alignment of the primary sequences of different retroviral INs has revealed that the C-terminal 28 amino acid tail of MoMLV IN is unique to the gammaretroviruses (112) (Figure 2D). For a long time, the functional significance of this tail had remained enigmatic, as various deletions of it did not significantly affect MoMLV infectivity (113). Recent reports have clarified that the role of the C-terminal tail is to directly bind the BET proteins (21,87,112). Nuclear magnetic resonance, MS-based protein footprinting and site-directed mutagenesis experiments have collectively identified that the MoMLV IN C-terminal tail (amino acid residues 386–405) directly mediates interactions with BET proteins (21,87,112). This region of MoMLV IN was disordered in the unliganded CTD structure but became ordered in the presence of the BRD3 ET domain (112). Importantly, C-terminal truncation mutants of recombinant MoMLV IN lacking all or part of the C-terminal tail exhibited markedly impaired interaction with BRD4, but retained wild-type levels of IN catalytic activities (87). Consistent with this observation, the MoMLV C-terminal deletion mutant 1–385 lost the ability to interact with BRD2, 3 and 4, and a 24-mer peptide composed of IN residues 386–408 (Figure 2D) disrupted the interaction between full length IN and the BRD3 ET domain *in vitro* (112). Somewhat unclear is the extent to which MoMLV IN regions outside the C-terminal tail might contribute to BET protein binding. Whereas Sharma *et al.* did not detect any binding to a CTD deletion mutant of MoMLV IN (19), Gupta *et al.* reported that an N-terminal deletion mutation that removed the NTD and first 50 residues of the CCD significantly reduced binding despite the fact that this construct harbored an intact C-terminal tail (20). Results of Ala-scanning mutagenesis led these investigators to suggest that residues that compose CCD α helix 6 contributed to BET protein binding (20). In summary, whereas numerous groups have highlighted the importance of the MoMLV IN C-terminal tail region in BET protein binding (19–21,87,112), additional work is required to help clarify the extent to which the CCD contributes to overall binding affinity.

There is precedence for integration-mediated targeting through the C-terminal tail of an IN protein and a cognate chromatin binding protein. Retrotransposons are analogous to retroviruses, with the exception that they lack an extracellular phase of replication. To avoid inactivation of essential genes, the integration of these elements is tightly linked to subsets of genomic loci. In the case of the yeast Ty5 retrotransposon, integration is favored into heterochromatin (114). Ty5 integration targeting is mediated by a 6-mer peptide at the IN C-terminus and the host heterochromatin protein Sir4p (115). With now two examples of retroelement integration targeting mediated between the C-terminal tail of IN and a cognate chromatin binding protein, we predict that other viruses/transposons will also be found to take advantage of this design to steer the integration of their reverse transcripts.

Recent truncation mutagenesis and MS-based protein footprinting experiments have identified the ET domain of BET proteins as the primary interface interacting with MoMLV IN (19–21,87). The BID region of BRD4 contributed additional interactions and increased the binding affinity to MoMLV IN (87). Complementary NMR and mutagenesis experiments have defined the MoMLV IN binding sites on the BRD4 ET domain in more detail (20,21,87). The majority of interacting residues are located on ET helices 2 and 3 and the loop connecting these two helices (Figure 2E). These studies have provided structural clues for the specificity and high affinity binding between BRD4 and MoMLV IN. The determination of the structure of the MoMLV IN CTD bound to a BRD ET domain is expected to provide further valuable details about how the viral and cellular proteins recognize each other.

Interestingly, whereas expression of the LEDGF/p75 IBD in target cells can potently inhibit HIV-1 infection and integration (67,69,70), over expressing the IBD of BRD2 (residues 640–801) stimulated MoMLV infection and integration ∼2-fold (20). Although the reason behind this rather dramatic difference is unclear, we speculate it may lie in the mode of host factor-IN binding. As discussed, the LEDGF/p75 IBD engages IN at the CCD-CCD dimer interface (108) (Figure 2C), which is also the binding site of a potent class of small molecule inhibitors that promote IN multimerization and inhibit HIV-1 IN catalysis *in vitro* (116–118). Conceivably, over expressed IBD protein in target cells, which functionally inhibits viral DNA integration, could similarly inhibit IN catalysis. BET proteins by contrast engage a functionally inert aspect of gammaretroviral IN structure, the disordered C-terminal tail (87,112). Accordingly, we conclude that forced expression of a BET protein IBD in target cells is unlikely to deregulate IN catalytic function. The purified ET domains of BRD2, 3 and 4 could moreover stimulate MoMLV IN strand transfer activity *in vitro* (20), indicating that the protein domains might have similar IN stimulatory activity during virus infection. Consistent with this interpretation, immunoprecipitation of ectopically expressed green fluorescent protein fusions to either BRD2 or BRD4 co-precipitated IN from cells infected with MoMLV (20). The analysis of HIV-1 and MoMLV PICs derived from cells over expressing the LEDGF/p75 or BET protein IBD should reveal if different levels of IN catalytic function determine the differences observed in viral titer under these infection conditions.

## LEDGF/p75 AND BET PROTEINS NAVIGATE RETROVIRAL PICs TO SELECT CHROMATIN MARKS

**Figure 3.**
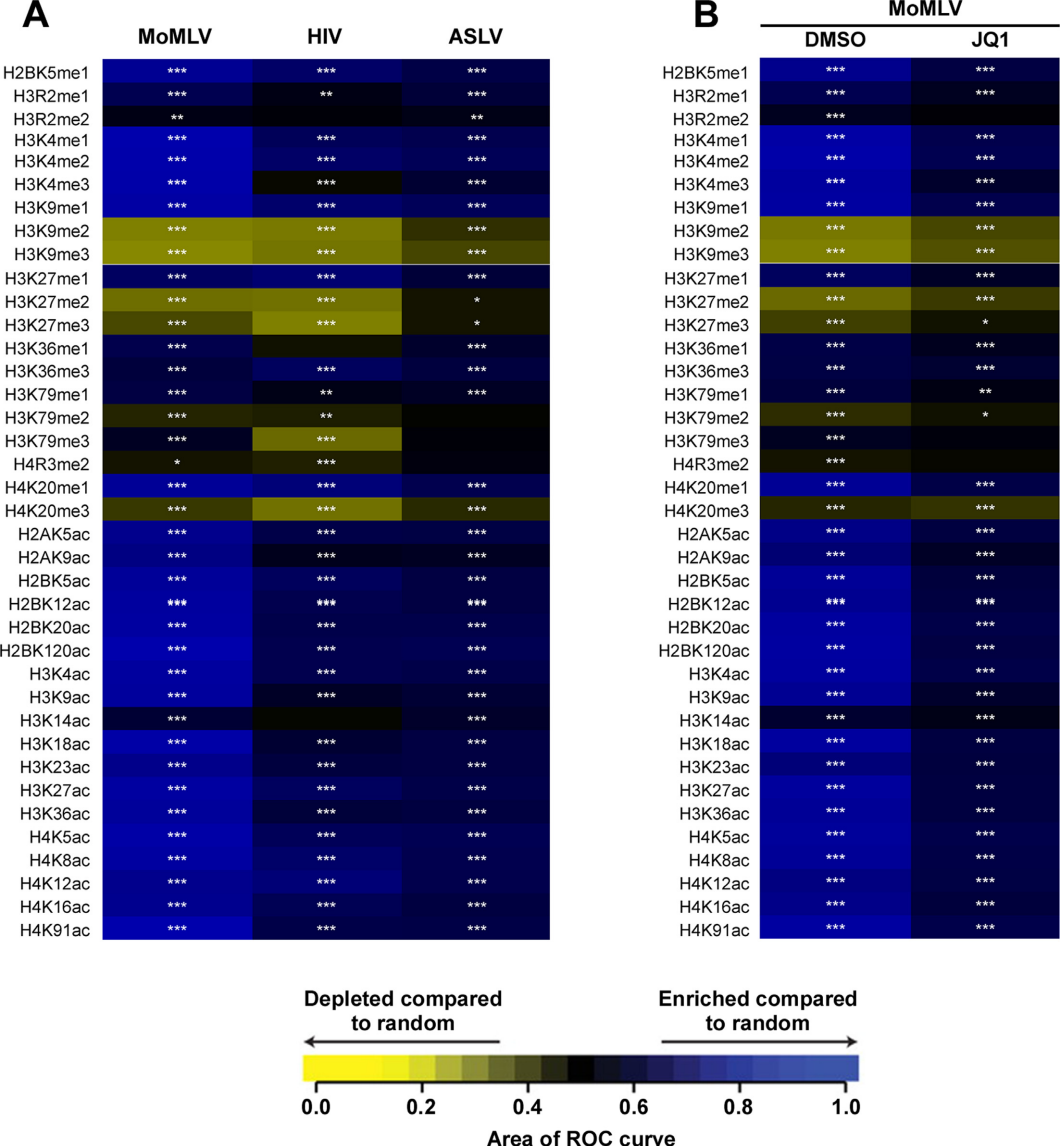
Heatmaps depicting relationships between retroviral integration frequencies and histone post-translational modifications. For both panels (**A** and **B**), the integration site data sets are shown in columns with the histone post-translational modifications in the rows labeled to the left. The relationship between the integration site frequencies relative to matched random controls for each of the annotated histone post-translational modification was quantified by the receiver operator characteristic (ROC) curve area method. The color key depicts enrichment or depletion of the annotated feature near integration sites. *P*-values are for individual integration site datasets compared to match random controls, ***P < 0.001; **P < 0.01; *P < 0.05. (A) Integration frequencies of different retroviruses including MoMLV, HIV-1 and ASLV. (B) Integration frequencies of MoMLV with respect to histone post-translational modifications following treatment with either DMSO or the JQ-1 (500 nM) inhibitor. Figure adapted from (87).

The tethering roles of LEDGF/p75 and BET proteins imply that the interactions of these cellular proteins with cognate chromatin features in turn influences retroviral integration site selectivity. Indeed, LEDGF/p75 recognizes histone H3 tails containing trimethylated Lys36 (H3K36me3) (119–121), which is an epigenetic marker for active transcription units and positively correlates with HIV-1 integration sites (73,122; also see Figure 3A). On the other hand, BET proteins preferentially engage certain acetylated H3 and H4 peptides including H3K9ac, H3K14ac, H3K27ac, H4K5ac, H4K8ac, H4K12ac and H4K16ac (123–126) that are enriched near TSSs and proto-oncogenes, and are preferred sites for MLV integration (87,122; also see Figure 3).

The N-terminal PWWP domain is the key determinant for the site selective association of LEDGF/p75 with chromatin. HIV-1 integration sites in the presence of wild type LEDGF/p75 differed substantially from those generated in the presence of truncation mutants that lacked the PWWP domain (127). NMR structures of the LEDGF/p75 PWWP domain revealed two distinct functional interfaces: a well-defined hydrophobic pocket that interacts with the H3K36me3 histone tail, and an adjacent basic interface that non-specifically engages DNA (120,121). Interestingly, the LEDGF/p75 PWWP domain exhibited low binding affinities for both an isolated H3K36me3 peptide and for naked DNA, whereas it interacted tightly with mononucleosomes that contained a tri-methyl-lysine analogue at position 36 of H3. These results indicate that cooperative binding of LEDGF/p75 with both the H3K36me3 tail and nucleosomal DNA is essential for the tight and site-selective association of LEDGF/p75 with chromatin (120). Indeed, mutations introduced in either the hydrophobic pocket or the basic surface significantly compromised the ability of LEDGF/p75 to both associate with chromatin and stimulate HIV-1 integration (128). These findings collectively indicate that LEDGF/p75-mediated navigation of lentiviral PICs to actively transcribed genes provides IN with increased access to nucleosomal DNA, which are the favored sites for integration both *in vitro* and in infected cells (35,36,129).

Similar to HIV-1, MoMLV integration sites are periodically distributed on nucleosomal DNA along cellular chromosomes (122). Furthermore, a recent study has suggested that akin to LEDGF/p75, BET proteins engage both DNA wrapped around the histone core and their cognate epigenetic marks to tightly bind chromatin (87). For example, BRD4 bound purified native mononucleosomes with significantly higher affinity than either naked DNA or isolated acetylated peptides. The two N-terminal bromodomains of BET proteins have been shown to interact with a number of acetylated H3 and H4 peptides but not with their unmodified counterparts (123–125). Furthermore, peptides containing multiple acetylated sites were particularly favored (123,130). Yet, the tightest binding affinity reported to date, ∼3 μM, which was seen between BRD4 BD-I and a tetra-acetylated H4 substrate, is a comparatively weak interaction (130). Two conserved motifs, A and B, which are located adjacent to the bromodomains (Figure 1B), exhibit highly basic interfaces and contribute to BET protein binding to DNA. However, BRD4 interacted with naked DNA with a relatively low binding affinity (∼2 μM) compared with the much tighter binding (*K*_d_ ∼60 nM) detected with native mononucleosomes (87). Thus the cooperative binding to both cognate histone marks and nucleosomal DNA could be a generic mechanism employed by various chromatin tethers to allow their tight interactions with select regions of chromatin.

Recent reports that significantly extended the number of unique MoMLV integration sites analyzed (∼3.9 million) have yielded novel insight into the mechanism of MoMLV PIC targeting (8,9). For example, approximately half of all MoMLV integrations occurred within 1.6–2.0% of the human genome (8). Close examination revealed that strong enhancers and active promoters are superior predictors of MoMLV integration as compared to TSSs. Clustered transcription factor binding sites essentially comprise enhancer elements, which function to form a platform for transcriptional regulatory complex recruitment (reviewed in 131). In terms of MoMLV integration, the greatest enrichment was found in enhancers that are characterized by H3K4me1, H3K4me2, H3Kme3, H3K27ac and H3K9ac marks (8,9). However, the precise hierarchy of favored histone modifications varied among cell type (8,9), which likely recapitulates the observation that the activities of many enhancers are cell-type specific (132). Independent studies suggested that enhancers are the major source of BRD4-dependent transcriptional activation (133) and that genes that are regulated by strong enhancers are particularly sensitive to BET inhibition (134). Because BET proteins are unlikely to directly engage methylated histone tails, their association with strong enhancers could be mediated through direct interactions with H3K27ac and H3K9ac marks and/or with congregated heterologous transcription factors (134).

Although the specificity of favored enhancer-associated epigenetic mark can vary among cell type, a significant number, about one-third, of targeted H3K4me1 marks in CD4+ T cells and CD34+ hematopoietic stem cells overlapped (9). Therefore, the correlation of integration sites in one cell type to mapped positions of histone epigenetic mark in a second cell type can yield overall global patterns of MoMLV site selectivity in response to BET protein disruption, for example through targeted siRNA depletion of host factors or JQ-1 treatment (21,87) (Figure 3B). The observation that JQ-1 treatment or concurrent BET protein depletion significantly reduced MoMLV integration frequencies at sites associated with enhancer and promoter-associated histone marks is consistent with the BET protein-mediated tethering mechanism of MoMLV integration (9,21,87). As the genomic occupancies of BRD2, 3 and 4 are non identical (135), further experimentation that correlates particular BET protein binding sites and histone epigenetic marks across cell type will help to better understand the detailed mechanisms that underlie MoMLV integration site selectivity.

**Figure 4.**
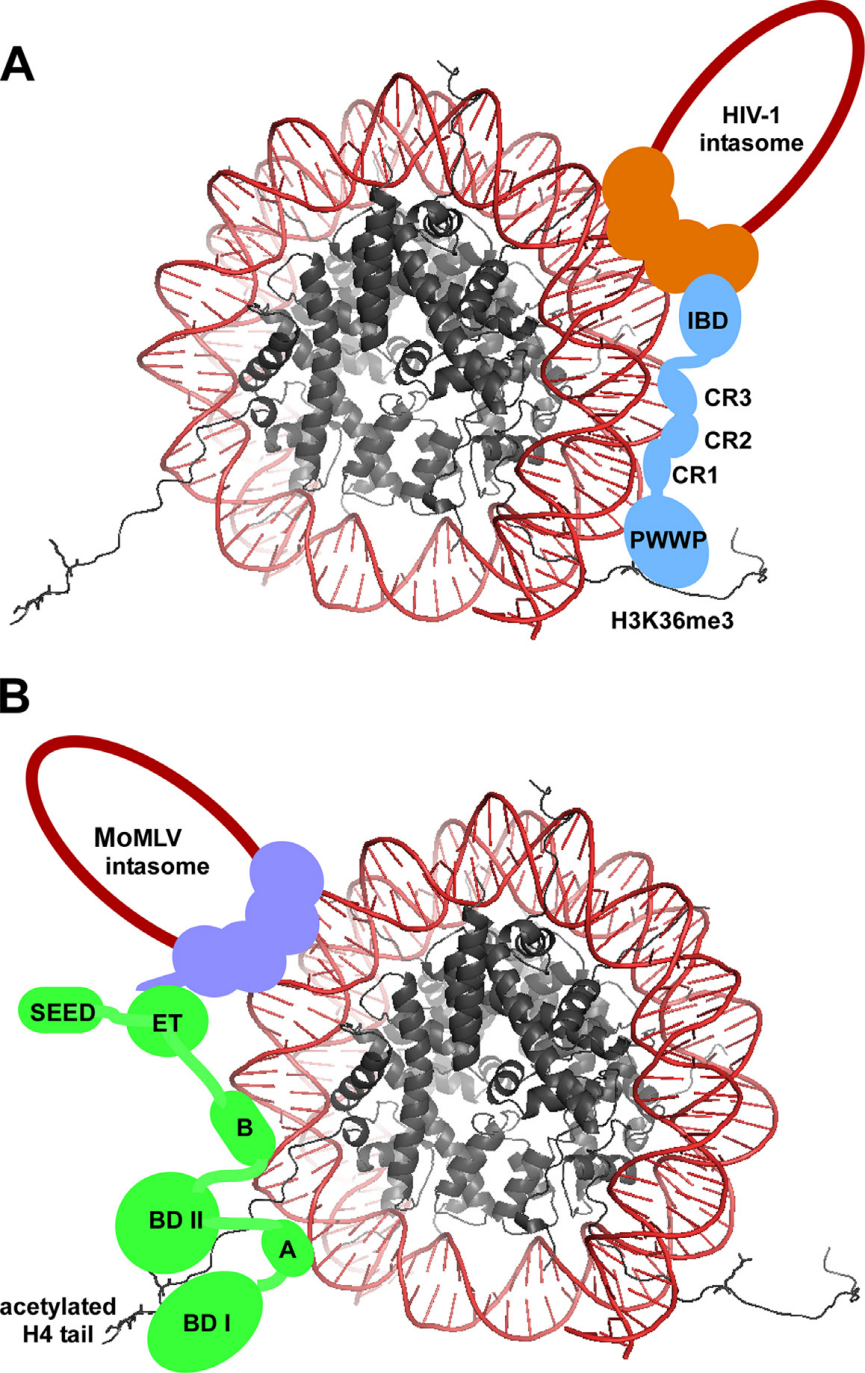
Model depicting the bimodal interactions of LEDGF/p75 and BET proteins with corresponding HIV-1 and MoMLV intasomes and mononucleosomes containing select histone marks. (**A**) LEDGF/p75 (depicted in blue) is able to bind selectively and with high affinity to mononucleosomes through the cooperative binding of the PWWP domain with the H3K36me3 histone tail and the three charge regions (CR1–3) with the DNA (shown in red) wrapped around the histones (shown in gray). The C-terminal IBD of LEDGF/p75 is able to directly engage the HIV-1 intasome (depicted with a tetramer of HIV-1 IN in orange and viral DNA, in a dark red single line). (**B**) A BRD protein (depicted in green) is able to bind selectively and with high affinity to mononucleosomes through the cooperative binding of the dual bromodomains with acetylated H3 and H4 histone tails (H4 acetylation depicted here) and motifs A and B with DNA (shown in red) wrapped around the histones (shown in gray). The C-terminal region of the BET protein is able to engage the MoMLV intasome (depicted with a tetramer of MoMLV IN in purple and viral DNA in a dark red single line) through its extra terminal (ET) domain, which binds to the C-terminal tail of MoMLV IN. The SEED domain does not directly contribute to these interactions but may play an accessory role in complex stability (87).

Despite the fact that LEDGF/p75 and BET proteins recognize distinct histone marks and bind different retroviral INs, the overall bimodal interaction (Figure 4) used to tether retroviral PICs to chromatin seems to be a common mechanism. HIV-1 and MoMLV depend on these cellular factors for effective and timely access for the integration of their viral DNAs into host chromosomes. Relatively rapid targeting to chromatin acceptor sites for IN-mediated strand transfer is likely crucial for virus survival, as the propensity for unintegrated DNA to be either degraded or modified by cellular proteins increases over time. For example, the two viral DNA ends can be ligated to form 2-long terminal repeat (LTR)-containing circles, which are a dead end for the viruses because they cannot support replication (136). Another key reason for HIV-1 and MoMLV to utilize LEDGF/p75 and BET proteins is to preferentially position their viral DNA into transcriptionally active regions of the host genome. Having such a distribution of proviral DNA should facilitate viral gene expression. Therefore, the ability of LEDGF/p75 and BET proteins to both enhance integration efficiency and preferentially target the site of integration into favorable regions for HIV-1 and MoMLV collectively ensures for effective viral replication.

## OTHER VIRAL AND CELLULAR FACTORS AFFECTING INTEGRATION SITE SELECTIVITY

HIV-1 and MoMLV take different paths en route to the host chromosomal sites for integration. Lentiviruses can efficiently infect non-dividing cells, and their PICs can accordingly traverse through the nuclear pore complexes that perforate the interphase nuclear envelope (137 for review). MoMLV PICs lack this ability, and gammaretroviruses accordingly rely on mitosis and nuclear envelope dissolution to access cell chromosomes (138). Therefore, it is not surprising that a number of cellular proteins that are involved in nuclear transport have also been shown to influence HIV-1 but not MoMLV integration. The key HIV-1 determinant that governs PIC nuclear import is the viral capsid (CA) protein, which is expressed as part of the Gag structural precursor protein (137,139).

Genome-wide siRNA screens have identified cell host factors that are important for efficient HIV-1 infection (140–142). Of these hits, nucleoporin (NUP) proteins NUP358 (also known as RanBP2) and NUP153, as well as the beta-karyopherin transportin-3/TNPO3 (also known as TRN-SR2), have been scrutinized for their roles in the early steps of HIV-1 replication (143–148). Depletion of these cellular proteins could not only adversely affect PIC nuclear localization and integration efficiency, but also alter the pattern of HIV-1 proviruses along chromatin. In particular, RanBP2, TNPO3 or NUP153 depletion resulted in reduced HIV-1 integration frequencies in gene dense regions of chromosomes (144,146,147). As noted earlier, IN is the key viral protein that governs integration site selection (13). To investigate potential roles for other viral proteins in integration site selection, chimeric HIV-1 viruses containing the substitution of MoMLV Gag counterparts were previously examined. Interestingly, these chimeric viruses displayed reduced integration frequencies in gene rich regions, which suggested a Gag-dependent role in integration site targeting (13,144,146). Moreover, a single missense mutation that resulted in an N74D change in HIV-1 CA counteracted the preference for HIV-1 to integrate into gene-rich regions of chromosomes (146,149). Because the mutant virus with an N74D CA substitution efficiently infected cells that were depleted for RanBP2, TNPO3 or NUP153, its novel integration profile may be linked to an alternative pathway of PIC nuclear import. Collectively, these findings suggest that the route taken by HIV-1 PICs during nuclear import is directly linked to integration site selection. Accordingly, a two-step model has been proposed, where during nuclear entry the nuclear pore components direct HIV-1 PICs toward regions of high gene density, after which the PICs engage LEDGF/p75 to gain access to active gene bodies (144,146). Consistent with this interpretation, results of fluorescent imaging indicate that HIV-1 has the propensity to integrate into chromatin that is associated with the nuclear periphery (150).

The interaction of MoMLV IN with BET proteins is the best studied example of a virus-host interaction that determines gammaretroviral integration target site selection. However, it is noteworthy that even with potent inhibition of BET proteins by JQ-1, which blocks their interactions with cognate histone marks, integration events at TSSs, while significantly reduced, were still substantially higher than random or when compared to HIV-1 (19,21). These findings suggest that additional host and/or viral factors could contribute to the integration pattern characteristic of MoMLV. One component of MoMLV PICs is the p12 Gag protein, which has been shown to mediate the association between PICs and condensed mitotic chromosomes (151). However, mutations that altered p12 interactions with chromatin had no detectable effects on MoMLV integration target site selection (152). Identification of new players in MoMLV integration target site selection will not only help to elucidate the molecular mechanisms of MoMLV integration, but will also inform ongoing efforts to develop retroviral vectors for human gene-therapy.

## IMPLICATIONS FOR DEVELOPING RETROVIRAL VECTORS FOR HUMAN GENE-THERAPY

Retroviral vectors have been successfully used in clinical human gene-therapy to rectify monogenic disorders by stably expressing the therapeutic transgene in patients. Replication-defective vectors have been derived from various retroviral genera, such as gammaretrovirus, lentivirus and spumavirus, as well as from retotransposons (reviewed in 153). The widespread success of first-generation gammaretrovirus-based vectors for human gene-therapy resulted from their use in the correction of primary immunodeficiencies, such as X-linked severe combined immunodeficiency (SCID-X1), adenosine deaminase-SCID (ADA-SCID), Wiskott–Aldrich syndrome (WAS) and X-linked chronic granulomatous disease (CGD) (reviewed in 154–157). The therapeutic concept for utilizing gammaretroviral MoMLV-based vectors was first successfully demonstrated for autologous hematopoietic stem cell (HSC) gene therapy for SCID-X1 (24). In separate clinical trials from 1999 to 2009, a total of 20 SCID-X1 patients underwent treatment for a gene defect in interleukin 2 common gamma chain (IL2γc) using MoMLV-based HSC gene therapy (158). Autologous CD34+ cells derived from patient bone marrow were transduced *ex vivo* with MoMLV-based vectors carrying the IL2γc transgene. Clinical benefits were achieved in 17 of 20 patients who displayed transgene expression, restoration of T-cell function and long-term immunological correction. Unfortunately, severe adverse events occurred in five of the 20 patients, who developed leukemia (158). The associated cancer in these patients was linked to the insertion of MoMLV-based vectors near the *LMO-2* proto-oncogene in four instances and near the *CCND2* proto-oncogene in the remaining case (26–28). The integrations led to MoMLV-LTR driven transcriptional upregulation of the nearby proto-oncogenes (26–28). In separate studies for the treatment of different genetic diseases, such as WAS and CGD, patients likewise have developed cancer (159–161).

The adverse outcomes from these clinical trials have highlighted the significance of exploring the molecular mechanisms of retroviral integration site selection for developing ‘safer’ retroviral vectors for human gene-therapy (162). The genotoxicity associated with retroviral vector integration in the host genome can be explained by the following mechanisms (also reviewed in 163–165): (i) activation of host gene promoters by enhancers present in the viral LTRs, leading to transcriptional activation and upregulation of host genes, (ii) transcriptional read-through of the host gene resulting in aberrant and/or chimeric transcripts whose expression can result in adverse effects and (iii) deregulation of host gene expression due to cryptic splicing or premature polyadenylation of host genes due to RNA elements present in the viral LTR. Interestingly, ∼0.12% of all MoMLV integration occurred in the vicinity of the *LMO-2* proto-oncogene in CD34+ cells whereas integration in this region was not detected in CD4+ cells (9). These observations highlight the utility of determining retroviral integration site distributions in clinically relevant target cells prior to *in vivo* transplantation.

In depth analysis of the SCID-X1 gene-therapy trial has established that the initiation of leukemia was due to the transcriptional activation of proto-oncogenes by MoMLV vector LTRs (26–28). Additionally, it was shown that the leukemic T cell clones accumulated secondary genetic aberrations such as translocations and deletions, consistent with the ‘multiple-hit’ hypothesis of oncogenesis (166,167). Thus, integration of retroviral vectors near proto-oncogenes or growth control genes can prime the transformation process and lead to the expansion of aberrant clones by clonal dominance. In light of these points, second-generation retroviral vectors have been developed. These self-inactivating (SIN) vectors bear deletions in the U3 region of the viral LTR, which contains the viral enhancer/promoter elements. The SIN vectors have displayed a safer profile in *in vitro* genotoxicity assays (168–170) and have been used in recent clinical trials for SCID-X1, ADA-SCID, WAS and X-CGD (171).

Recent identification of the key role of BET proteins for MoMLV integration site selectivity has opened up new paths to modulate gene-therapy applications with the goal to suppress unwanted genotoxicity. For example, JQ-1 treatment has been shown in a cell line model to reduce the frequency of MoMLV integration in the vicinity of proto-oncogenes (19,87). Accordingly, CD34+ cells could be treated with BET protein inhibitors during *ex vivo* transduction with MoMLV-based vectors, though initial work would need to determine integration frequencies near proto-oncogenes in comparison to previously utilized cell line models and additionally address any potential toxicity of the small molecules in CD34+ cells. Ongoing clinical trials to determine the safety profiles of second generation BET inhibitors such as I-BET762 (172) and OTX015 (173) in the treatment of human cancers should help inform as to which molecules could have utility with MoMLV-based gene-therapy vectors.

An alternative approach to counteract the genotoxicity of MoMLV-based vectors would be to utilize chimeric cellular proteins to redirect MoMLV integration away from proto-oncogenes and toward ‘safer’ chromosomal sites. For example, a proof of concept study showed that ectopically expressed LEDGF(1–324)/BRD4(ET/SEED) redirected MoMLV integration away from TSSs and toward active genes (21). However, the potential clinical applications of BET protein-mediated MoMLV retargeting are fairly unclear. One significant drawback is the requirement of having the chimeric tethering factor in the target cell, presumably in advance of challenge with the therapeutic retroviral vector. A more direct approach might be to utilize MoMLV IN C-terminal tail deletion mutant vectors. While various deletions or mutations of the MoMLV IN C-terminal tail markedly compromised IN binding to BET proteins (19,21,87,112), the terminal tail region is not essential for catalytic activities of the enzyme *in vitro* (174–176) or for virus replication in cell culture (113,177–179). MoMLV mutants with deleted or altered IN C-terminal tails displayed markedly reduced integration frequencies near TSSs, CpG islands and BET protein-binding sites (112). For example, integration frequencies for wild-type and mutant MLVs within 1 kb of TSSs averaged ∼12 and ∼2.5%, respectively. Yet, the residual preference of mutant MoMLVs for this chromatin region was still evident when compared to HIV-1. One potential explanation for this observation is the residual affinity of BET proteins to bind C-terminal truncation mutants of MoMLV IN (20). Alternatively, secondary chromatin-associated factors might arise in the absence of BET protein engagement, as occurs for HIV-1 in the absence of LEDGF/p75 protein (16,17).

The problems encountered with MoMLV-based vectors have prompted the development of HIV-derived lentiviral vectors. As discussed above, lentiviral vectors unlike MoMLV are able to transduce both dividing and non-dividing differentiated cells with high efficiency (180). Even more importantly, HIV-1 integration is disfavored near TSSs and proto-oncogenes, which could reduce the risks of transcriptional activation. In a recent clinical trial involving one patient, a lentiviral vector was successfully employed to correct beta thalassemia major (181). Interestingly, in this case the vector integrated within the *HMGA2* proto-oncogene, however the respective cell clone expanded without leukemic progression (181). Clinical trials with larger number of individuals will allow assessing the risks versus benefit ratios for the clinical utility of lentiviral vectors.

Electrostatic interactions between the HIV-1 IN NTD and LEDGF/p75 IBD, which are important for the high affinity interaction, have been scrutinized to artificially control HIV-1 integration site selectivity (109,182). Reverse-charged mutations were engineered at the interacting interfaces of both proteins to allow mutant HIV-1 IN to recognize complementary mutant LEDGF/p75, but not the respective wild-type counterparts. The transduction efficiency of an optimized mutant IN vector, which was reduced to ∼10–20% compared with the wild-type vector in cells expressing wild-type LEDGF/p75, increased to ∼75% upon ectopic expression of complementary reverse-engineered LEDGF/p75 (182). The application of this approach can be extended to develop heterologous fusion proteins containing the mutant LEDGF/p75 IBD and desired chromatin tethering modules to control the HIV-1 integration pattern. Nevertheless, customized lentiviral retargeting strategies suffer the common drawback of ectopic expression of the retargeting factor in susceptible target cells (182). Clinical trials could potentially compare modified MoMLV and HIV-1 based vectors to MMTV-based constructs, as this betaretrovirus reportedly targets host chromatin in a random fashion (11,12).

## SUMMARY

Recent research has clarified the molecular mechanisms that underlie integration site selection of retroviruses. The propensity for weakly conserved palindromic sequences at the sites of integration seemingly reflects IN-target DNA interactions that preferentially bend the DNA to position it near the two IN active sites within the functional intasome complex. Of the six profiled retroviral genera—the genome-wide distribution of epsilonretrovirus integration has yet to be reported—gammaretroviruses and lentiviruses display the most distinctive profiles. Whereas the interaction between LEDGF/p75 and HIV-1 IN targets integration into active gene bodies, MoMLV IN engages BET proteins to integrate in the vicinity of strong enhancers and the TSSs of active gene promoters. Though many differences exist between these two systems, including the regions of the IN protein that interact with its cognate host cellular protein and the resulting epigenetic mark to which the intasome complex is tethered, the overall concept of bimodal tethering of PIC-associated IN to specific regions of chromatin is strikingly similar and parallel findings in the related area of retrotransposon integration targeting. For retroviruses these interactions likely help to situate the provirus within well-expressed regions of the host genome. These discoveries have sprung novel initiatives toward controlling the specificity of retroviral DNA integration, in particular for the field of human gene-therapy. For example, the potential targeting of MoMLV vectors away from TSSs and oncogenes by generating clinical vectors that lack the C-terminal tail of MoMLV IN and hence do not engage BET proteins may prove less genotoxic than previous MoMLV-based clinical vectors. The field of retroviral integration site targeting is quickly evolving, with exciting advances on the basic mechanism and utility of virus-derived vectors for treating human genetic disorders expectedly forthcoming.

## References

[1] Bushman F.D. (2007). Retroviral integration and human gene therapy. J. Clin. Invest..

[2] Engelman A. (1994). Most of the avian genome appears available for retroviral DNA integration. Bioessays.

[3] Carteau S., Hoffmann C., Bushman F. (1998). Chromosome structure and human immunodeficiency virus type 1 cDNA integration: centromeric alphoid repeats are a disfavored target. J. Virol..

[4] Derse D., Crise B., Li Y., Princler G., Lum N., Stewart C., McGrath C.F., Hughes S.H., Munroe D.J., Wu X. (2007). Human T-cell leukemia virus type 1 integration target sites in the human genome: comparison with those of other retroviruses. J. Virol..

[5] Cavazza A., Moiani A., Mavilio F. (2013). Mechanisms of retroviral integration and mutagenesis. Hum. Gene Ther..

[6] Schroder A.R., Shinn P., Chen H., Berry C., Ecker J.R., Bushman F. (2002). HIV-1 integration in the human genome favors active genes and local hotspots. Cell.

[7] Wu X., Li Y., Crise B., Burgess S.M. (2003). Transcription start regions in the human genome are favored targets for MLV integration. Science.

[8] LaFave M.C., Varshney G.K., Gildea D.E., Wolfsberg T.G., Baxevanis A.D., Burgess S.M. (2014). MLV integration site selection is driven by strong enhancers and active promoters. Nucleic Acids Res..

[9] De Ravin S.S., Su L., Theobald N., Choi U., Macpherson J.L., Poidinger M., Symonds G., Pond S.M., Ferris A.L., Hughes S.H. (2014). Enhancers are major targets for murine leukemia virus vector integration. J. Virol..

[10] Narezkina A., Taganov K.D., Litwin S., Stoyanova R., Hayashi J., Seeger C., Skalka A.M., Katz R.A. (2004). Genome-wide analyses of avian sarcoma virus integration sites. J. Virol..

[11] Faschinger A., Rouault F., Sollner J., Lukas A., Salmons B., Gunzburg W.H., Indik S. (2008). Mouse mammary tumor virus integration site selection in human and mouse genomes. J. Virol..

[12] Konstantoulas C.J., Indik S. (2014). Mouse mammary tumor virus-based vector transduces non-dividing cells, enters the nucleus via a TNPO3-independent pathway and integrates in a less biased fashion than other retroviruses. Retrovirology.

[13] Lewinski M.K., Yamashita M., Emerman M., Ciuffi A., Marshall H., Crawford G., Collins F., Shinn P., Leipzig J., Hannenhalli S. (2006). Retroviral DNA integration: viral and cellular determinants of target-site selection. PLoS Pathog..

[14] Ciuffi A., Bushman F.D. (2006). Retroviral DNA integration: HIV and the role of LEDGF/p75. Trends Genet..

[15] Ciuffi A., Llano M., Poeschla E., Hoffmann C., Leipzig J., Shinn P., Ecker J.R., Bushman F. (2005). A role for LEDGF/p75 in targeting HIV DNA integration. Nat. Med..

[16] Marshall H.M., Ronen K., Berry C., Llano M., Sutherland H., Saenz D., Bickmore W., Poeschla E., Bushman F.D. (2007). Role of PSIP1/LEDGF/p75 in lentiviral infectivity and integration targeting. PLoS One.

[17] Shun M.C., Raghavendra N.K., Vandegraaff N., Daigle J.E., Hughes S., Kellam P., Cherepanov P., Engelman A. (2007). LEDGF/p75 functions downstream from preintegration complex formation to effect gene-specific HIV-1 integration. Genes Dev..

[18] Schrijvers R., De Rijck J., Demeulemeester J., Adachi N., Vets S., Ronen K., Christ F., Bushman F.D., Debyser Z., Gijsbers R. (2012). LEDGF/p75-independent HIV-1 replication demonstrates a role for HRP-2 and remains sensitive to inhibition by LEDGINs. PLoS Pathog..

[19] Sharma A., Larue R.C., Plumb M.R., Malani N., Male F., Slaughter A., Kessl J.J., Shkriabai N., Coward E., Aiyer S.S. (2013). BET proteins promote efficient murine leukemia virus integration at transcription start sites. Proc. Natl. Acad. Sci. U.S.A..

[20] Gupta S.S., Maetzig T., Maertens G.N., Sharif A., Rothe M., Weidner-Glunde M., Galla M., Schambach A., Cherepanov P., Schulz T.F. (2013). Bromo- and extraterminal domain chromatin regulators serve as cofactors for murine leukemia virus integration. J. Virol..

[21] De Rijck J., de Kogel C., Demeulemeester J., Vets S., El Ashkar S., Malani N., Bushman F.D., Landuyt B., Husson S.J., Busschots K. (2013). The BET family of proteins targets Moloney murine leukemia virus integration near transcription start sites. Cell Rep..

[22] Burns J.C., Friedmann T., Driever W., Burrascano M., Yee J.K. (1993). Vesicular stomatitis virus G glycoprotein pseudotyped retroviral vectors: concentration to very high titer and efficient gene transfer into mammalian and nonmammalian cells. Proc. Natl. Acad. Sci. U.S.A..

[23] Kafri T. (2004). Gene delivery by lentivirus vectors an overview. Methods Mol. Biol..

[24] Cavazzana-Calvo M., Hacein-Bey S., de Saint Basile G., Gross F., Yvon E., Nusbaum P., Selz F., Hue C., Certain S., Casanova J.L. (2000). Gene therapy of human severe combined immunodeficiency (SCID)-X1 disease. Science.

[25] Hacein-Bey-Abina S., von Kalle C., Schmidt M., Le Deist F., Wulffraat N., McIntyre E., Radford I., Villeval J.L., Fraser C.C., Cavazzana-Calvo M. (2003). A serious adverse event after successful gene therapy for X-linked severe combined immunodeficiency. N. Engl. J. Med..

[26] Schmidt M., Zickler P., Hoffmann G., Haas S., Wissler M., Muessig A., Tisdale J.F., Kuramoto K., Andrews R.G., Wu T. (2002). Polyclonal long-term repopulating stem cell clones in a primate model. Blood.

[27] Hacein-Bey-Abina S., Von Kalle C., Schmidt M., McCormack M.P., Wulffraat N., Leboulch P., Lim A., Osborne C.S., Pawliuk R., Morillon E. (2003). LMO2-associated clonal T cell proliferation in two patients after gene therapy for SCID-X1. Science.

[28] Hacein-Bey-Abina S., Garrigue A., Wang G.P., Soulier J., Lim A., Morillon E., Clappier E., Caccavelli L., Delabesse E., Beldjord K. (2008). Insertional oncogenesis in 4 patients after retrovirus-mediated gene therapy of SCID-X1. J. Clin. Invest..

[29] Bushman F.D., Fujiwara T., Craigie R. (1990). Retroviral DNA integration directed by HIV integration protein in vitro. Science.

[30] Engelman A., Mizuuchi K., Craigie R. (1991). HIV-1 DNA integration: mechanism of viral DNA cleavage and DNA strand transfer. Cell.

[31] Maertens G.N., Hare S., Cherepanov P. (2010). The mechanism of retroviral integration from X-ray structures of its key intermediates. Nature.

[32] Hare S., Maertens G.N., Cherepanov P. (2012). 3′-processing and strand transfer catalysed by retroviral integrase in crystallo. EMBO J..

[33] Pryciak P.M., Sil A., Varmus H.E. (1992). Retroviral integration into minichromosomes in vitro. EMBO J..

[34] Pryciak P.M., Varmus H.E. (1992). Nucleosomes, DNA-binding proteins, and DNA sequence modulate retroviral integration target site selection. Cell.

[35] Pruss D., Reeves R., Bushman F.D., Wolffe A.P. (1994). The influence of DNA and nucleosome structure on integration events directed by HIV integrase. J. Biol. Chem..

[36] Pruss D., Bushman F.D., Wolffe A.P. (1994). Human immunodeficiency virus integrase directs integration to sites of severe DNA distortion within the nucleosome core. Proc. Natl. Acad. Sci. U.S.A..

[37] Katz R.A., Gravuer K., Skalka A.M. (1998). A preferred target DNA structure for retroviral integrase in vitro. J. Biol. Chem..

[38] Stevens S.W., Griffith J.D. (1996). Sequence analysis of the human DNA flanking sites of human immunodeficiency virus type 1 integration. J. Virol..

[39] Holman A.G., Coffin J.M. (2005). Symmetrical base preferences surrounding HIV-1, avian sarcoma/leukosis virus, and murine leukemia virus integration sites. Proc. Natl. Acad. Sci. U.S.A..

[40] Wu X., Li Y., Crise B., Burgess S.M., Munroe D.J. (2005). Weak palindromic consensus sequences are a common feature found at the integration target sites of many retroviruses. J. Virol..

[41] Berry C., Hannenhalli S., Leipzig J., Bushman F.D. (2006). Selection of target sites for mobile DNA integration in the human genome. PLoS Comput. Biol..

[42] Valkov E., Gupta S.S., Hare S., Helander A., Roversi P., McClure M., Cherepanov P. (2009). Functional and structural characterization of the integrase from the prototype foamy virus. Nucleic Acids Res..

[43] Johnson R.C., Stella S., Heiss J.K., Rice PA, Correll CC (2008). Protein-Nucleic Acid Interactions: Structural Biology.

[44] Li X., Krishnan L., Cherepanov P., Engelman A. (2011). Structural biology of retroviral DNA integration. Virology.

[45] Serrao E., Krishnan L., Shun M.C., Li X., Cherepanov P., Engelman A., Maertens G.N. (2014). Integrase residues that determine nucleotide preferences at sites of HIV-1 integration: implications for the mechanism of target DNA binding. Nucleic Acids Res..

[46] Devroe E., Silver P.A. (2002). Retrovirus-delivered siRNA. BMC Biotechnol..

[47] Cherepanov P., Maertens G., Proost P., Devreese B., Van Beeumen J., Engelborghs Y., De Clercq E., Debyser Z. (2003). HIV-1 integrase forms stable tetramers and associates with LEDGF/p75 protein in human cells. J. Biol. Chem..

[48] Turlure F., Devroe E., Silver P.A., Engelman A. (2004). Human cell proteins and human immunodeficiency virus DNA integration. Front. Biosci..

[49] Emiliani S., Mousnier A., Busschots K., Maroun M., Van Maele B., Tempe D., Vandekerckhove L., Moisant F., Ben-Slama L., Witvrouw M. (2005). Integrase mutants defective for interaction with LEDGF/p75 are impaired in chromosome tethering and HIV-1 replication. J. Biol. Chem..

[50] Maertens G., Cherepanov P., Pluymers W., Busschots K., De Clercq E., Debyser Z., Engelborghs Y. (2003). LEDGF/p75 is essential for nuclear and chromosomal targeting of HIV-1 integrase in human cells. J. Biol. Chem..

[51] Llano M., Delgado S., Vanegas M., Poeschla E.M. (2004). Lens epithelium-derived growth factor/p75 prevents proteasomal degradation of HIV-1 integrase. J. Biol. Chem..

[52] Ge H., Si Y., Roeder R.G. (1998). Isolation of cDNAs encoding novel transcription coactivators p52 and p75 reveals an alternate regulatory mechanism of transcriptional activation. EMBO J..

[53] Hendrix J., Gijsbers R., De Rijck J., Voet A., Hotta J., McNeely M., Hofkens J., Debyser Z., Engelborghs Y. (2011). The transcriptional co-activator LEDGF/p75 displays a dynamic scan-and-lock mechanism for chromatin tethering. Nucleic Acids Res..

[54] Maertens G.N., Cherepanov P., Engelman A. (2006). Transcriptional co-activator p75 binds and tethers the Myc-interacting protein JPO2 to chromatin. J. Cell Sci..

[55] Bartholomeeusen K., De Rijck J., Busschots K., Desender L., Gijsbers R., Emiliani S., Benarous R., Debyser Z., Christ F. (2007). Differential interaction of HIV-1 integrase and JPO2 with the C terminus of LEDGF/p75. J. Mol. Biol..

[56] Hughes S., Jenkins V., Dar M.J., Engelman A., Cherepanov P. (2010). Transcriptional co-activator LEDGF interacts with Cdc7-activator of S-phase kinase (ASK) and stimulates its enzymatic activity. J. Biol. Chem..

[57] Bartholomeeusen K., Christ F., Hendrix J., Rain J.C., Emiliani S., Benarous R., Debyser Z., Gijsbers R., De Rijck J. (2009). Lens epithelium-derived growth factor/p75 interacts with the transposase-derived DDE domain of PogZ. J. Biol. Chem..

[58] Yokoyama A., Cleary M.L. (2008). Menin critically links MLL proteins with LEDGF on cancer-associated target genes. Cancer Cell.

[59] Llano M., Vanegas M., Hutchins N., Thompson D., Delgado S., Poeschla E.M. (2006). Identification and characterization of the chromatin-binding domains of the HIV-1 integrase interactor LEDGF/p75. J. Mol. Biol..

[60] Turlure F., Maertens G., Rahman S., Cherepanov P., Engelman A. (2006). A tripartite DNA-binding element, comprised of the nuclear localization signal and two AT-hook motifs, mediates the association of LEDGF/p75 with chromatin in vivo. Nucleic Acids Res..

[61] Cherepanov P., Devroe E., Silver P.A., Engelman A. (2004). Identification of an evolutionarily conserved domain in human lens epithelium-derived growth factor/transcriptional co-activator p75 (LEDGF/p75) that binds HIV-1 integrase. J. Biol. Chem..

[62] Llano M., Vanegas M., Fregoso O., Saenz D., Chung S., Peretz M., Poeschla E.M. (2004). LEDGF/p75 determines cellular trafficking of diverse lentiviral but not murine oncoretroviral integrase proteins and is a component of functional lentiviral preintegration complexes. J. Virol..

[63] Busschots K., Vercammen J., Emiliani S., Benarous R., Engelborghs Y., Christ F., Debyser Z. (2005). The interaction of LEDGF/p75 with integrase is lentivirus-specific and promotes DNA binding. J. Biol. Chem..

[64] Cherepanov P. (2007). LEDGF/p75 interacts with divergent lentiviral integrases and modulates their enzymatic activity in vitro. Nucleic Acids Res..

[65] Vandekerckhove L., Christ F., Van Maele B., De Rijck J., Gijsbers R., Van den Haute C., Witvrouw M., Debyser Z. (2006). Transient and stable knockdown of the integrase cofactor LEDGF/p75 reveals its role in the replication cycle of human immunodeficiency virus. J. Virol..

[66] Zielske S.P., Stevenson M. (2006). Modest but Reproducible Inhibition of Human Immunodeficiency Virus Type 1 Infection in Macrophages following LEDGFp75 Silencing. J. Virol..

[67] Llano M., Saenz D.T., Meehan A., Wongthida P., Peretz M., Walker W.H., Teo W., Poeschla E.M. (2006). An essential role for LEDGF/p75 in HIV integration. Science.

[68] Fadel H.J., Morrison J.H., Saenz D.T., Fuchs J.R., Kvaratskhelia M., Ekker S.C., Poeschla E.M. (2014). TALEN knockout of the PSIP1 gene in human cells: Analyses of HIV-1 replication and allosteric integrase inhibitor mechanism. J. Virol..

[69] De Rijck J., Vandekerckhove L., Gijsbers R., Hombrouck A., Hendrix J., Vercammen J., Engelborghs Y., Christ F., Debyser Z. (2006). Overexpression of the lens epithelium-derived growth factor/p75 integrase binding domain inhibits human immunodeficiency virus replication. J. Virol..

[70] Meehan A.M., Saenz D.T., Morrison J., Hu C., Peretz M., Poeschla E.M. (2011). LEDGF dominant interference proteins demonstrate prenuclear exposure of HIV-1 integrase and synergize with LEDGF depletion to destroy viral infectivity. J. Virol..

[71] Meehan A.M., Saenz D.T., Morrison J.H., Garcia-Rivera J.A., Peretz M., Llano M., Poeschla E.M. (2009). LEDGF/p75 proteins with alternative chromatin tethers are functional HIV-1 cofactors. PLoS Pathog..

[72] Ferris A.L., Wu X., Hughes C.M., Stewart C., Smith S.J., Milne T.A., Wang G.G., Shun M.C., Allis C.D., Engelman A. (2010). Lens epithelium-derived growth factor fusion proteins redirect HIV-1 DNA integration. Proc. Natl. Acad. Sci. U.S.A..

[73] Gijsbers R., Ronen K., Vets S., Malani N., De Rijck J., McNeely M., Bushman F.D., Debyser Z. (2010). LEDGF hybrids efficiently retarget lentiviral integration into heterochromatin. Mol. Ther..

[74] Silvers R.M., Smith J.A., Schowalter M., Litwin S., Liang Z., Geary K., Daniel R. (2010). Modification of integration site preferences of an HIV-1-based vector by expression of a novel synthetic protein. Hum. Gene Ther..

[75] De Rijck J., Bartholomeeusen K., Ceulemans H., Debyser Z., Gijsbers R. (2010). High-resolution profiling of the LEDGF/p75 chromatin interaction in the ENCODE region. Nucleic Acids Res..

[76] Pandey K.K., Sinha S., Grandgenett D.P. (2007). Transcriptional coactivator LEDGF/p75 modulates human immunodeficiency virus type 1 integrase-mediated concerted integration. J. Virol..

[77] McKee C.J., Kessl J.J., Shkriabai N., Dar M.J., Engelman A., Kvaratskhelia M. (2008). Dynamic modulation of HIV-1 integrase structure and function by cellular lens epithelium-derived growth factor (LEDGF) protein. J. Biol. Chem..

[78] Vanegas M., Llano M., Delgado S., Thompson D., Peretz M., Poeschla E. (2005). Identification of the LEDGF/p75 HIV-1 integrase-interaction domain and NLS reveals NLS-independent chromatin tethering. J. Cell Sci..

[79] Vandegraaff N., Devroe E., Turlure F., Silver P.A., Engelman A. (2006). Biochemical and genetic analyses of integrase-interacting proteins lens epithelium-derived growth factor (LEDGF)/p75 and hepatoma-derived growth factor related protein 2 (HRP2) in preintegration complex function and HIV-1 replication. Virology.

[80] Wang H., Jurado K.A., Wu X., Shun M.C., Li X., Ferris A.L., Smith S.J., Patel P.A., Fuchs J.R., Cherepanov P. (2012). HRP2 determines the efficiency and specificity of HIV-1 integration in LEDGF/p75 knockout cells but does not contribute to the antiviral activity of a potent LEDGF/p75-binding site integrase inhibitor. Nucleic Acids Res..

[81] Schrijvers R., Vets S., De Rijck J., Malani N., Bushman F.D., Debyser Z., Gijsbers R. (2012). HRP-2 determines HIV-1 integration site selection in LEDGF/p75 depleted cells. Retrovirology.

[82] Studamire B., Goff S.P. (2008). Host proteins interacting with the Moloney murine leukemia virus integrase: multiple transcriptional regulators and chromatin binding factors. Retrovirology.

[83] Wu S.Y., Chiang C.M. (2007). The double bromodomain-containing chromatin adaptor Brd4 and transcriptional regulation. J. Biol. Chem..

[84] Belkina A.C., Denis G.V. (2012). BET domain co-regulators in obesity, inflammation and cancer. Nat. Rev. Cancer.

[85] Moriniere J., Rousseaux S., Steuerwald U., Soler-Lopez M., Curtet S., Vitte A.L., Govin J., Gaucher J., Sadoul K., Hart D.J. (2009). Cooperative binding of two acetylation marks on a histone tail by a single bromodomain. Nature.

[86] Filippakopoulos P., Qi J., Picaud S., Shen Y., Smith W.B., Fedorov O., Morse E.M., Keates T., Hickman T.T., Felletar I. (2010). Selective inhibition of BET bromodomains. Nature.

[87] Larue R.C., Plumb M.R., Crowe B.L., Shkriabai N., Sharma A., Difiore J., Malani N., Aiyer S.S., Roth M.J., Bushman F.D. (2014). Bimodal high-affinity association of Brd4 with murine leukemia virus integrase and mononucleosomes. Nucleic Acids Res..

[88] Wu S.Y., Lee A.Y., Lai H.T., Zhang H., Chiang C.M. (2013). Phospho switch triggers Brd4 chromatin binding and activator recruitment for gene-specific targeting. Mol. Cell.

[89] Bisgrove D.A., Mahmoudi T., Henklein P., Verdin E. (2007). Conserved P-TEFb-interacting domain of BRD4 inhibits HIV transcription. Proc. Natl. Acad. Sci. U.S.A..

[90] Zhu J., Gaiha G.D., John S.P., Pertel T., Chin C.R., Gao G., Qu H., Walker B.D., Elledge S.J., Brass A.L. (2012). Reactivation of latent HIV-1 by inhibition of BRD4. Cell Rep..

[91] Boehm D., Calvanese V., Dar R.D., Xing S., Schroeder S., Martins L., Aull K., Li P.C., Planelles V., Bradner J.E. (2012). BET bromodomain-targeting compounds reactivate HIV from latency via a Tat-independent mechanism. Cell Cycle.

[92] Weidner-Glunde M., Ottinger M., Schulz T.F. (2010). WHAT do viruses BET on. Front. Biosci..

[93] You J., Croyle J.L., Nishimura A., Ozato K., Howley P.M. (2004). Interaction of the bovine papillomavirus E2 protein with Brd4 tethers the viral DNA to host mitotic chromosomes. Cell.

[94] Baxter M.K., McPhillips M.G., Ozato K., McBride A.A. (2005). The mitotic chromosome binding activity of the papillomavirus E2 protein correlates with interaction with the cellular chromosomal protein, Brd4. J. Virol..

[95] Banerjee C., Archin N., Michaels D., Belkina A.C., Denis G.V., Bradner J., Sebastiani P., Margolis D.M., Montano M. (2012). BET bromodomain inhibition as a novel strategy for reactivation of HIV-1. J. Leukoc. Biol..

[96] Li Z., Guo J., Wu Y., Zhou Q. (2013). The BET bromodomain inhibitor JQ1 activates HIV latency through antagonizing Brd4 inhibition of Tat-transactivation. Nucleic Acids Res..

[97] Wu S.Y., Lee A.Y., Hou S.Y., Kemper J.K., Erdjument-Bromage H., Tempst P., Chiang C.M. (2006). Brd4 links chromatin targeting to HPV transcriptional silencing. Genes Dev..

[98] Olejnik-Schmidt A.K., Schmidt M.T., Kedzia W., Gozdzicka-Jozefiak A. (2008). Search for cellular partners of human papillomavirus type 16 E2 protein. Arch. Virol..

[99] Chiang C.M. (2009). Brd4 engagement from chromatin targeting to transcriptional regulation: selective contact with acetylated histone H3 and H4. F1000 Biol. Rep..

[100] Leroy G., Chepelev I., Dimaggio P.A., Blanco M.A., Zee B.M., Zhao K., Garcia B.A. (2012). Proteogenomic characterization and mapping of nucleosomes decoded by Brd and HP1 proteins. Genome Biol..

[101] Nicodeme E., Jeffrey K.L., Schaefer U., Beinke S., Dewell S., Chung C.W., Chandwani R., Marazzi I., Wilson P., Coste H. (2010). Suppression of inflammation by a synthetic histone mimic. Nature.

[102] Zheng R., Jenkins T.M., Craigie R. (1996). Zinc folds the N-terminal domain of HIV-1 integrase, promotes multimerization, and enhances catalytic activity. Proc. Natl. Acad. Sci. U.S.A..

[103] Hare S., Gupta S.S., Valkov E., Engelman A., Cherepanov P. (2010). Retroviral intasome assembly and inhibition of DNA strand transfer. Nature.

[104] Krishnan L., Li X., Naraharisetty H.L., Hare S., Cherepanov P., Engelman A. (2010). Structure-based modeling of the functional HIV-1 intasome and its inhibition. Proc. Natl. Acad. Sci. U.S.A..

[105] Kessl J.J., Li M., Ignatov M., Shkriabai N., Eidahl J.O., Feng L., Musier-Forsyth K., Craigie R., Kvaratskhelia M. (2011). FRET analysis reveals distinct conformations of IN tetramers in the presence of viral DNA or LEDGF/p75. Nucleic Acids Res..

[106] Johnson B.C., Metifiot M., Ferris A., Pommier Y., Hughes S.H. (2013). A homology model of HIV-1 integrase and analysis of mutations designed to test the model. J. Mol. Biol..

[107] DeAnda F., Hightower K.E., Nolte R.T., Hattori K., Yoshinaga T., Kawasuji T., Underwood M.R. (2013). Dolutegravir interactions with HIV-1 integrase-DNA: structural rationale for drug resistance and dissociation kinetics. PLoS One.

[108] Cherepanov P., Ambrosio A.L., Rahman S., Ellenberger T., Engelman A. (2005). Structural basis for the recognition between HIV-1 integrase and transcriptional coactivator p75. Proc. Natl. Acad. Sci. U.S.A..

[109] Hare S., Shun M.C., Gupta S.S., Valkov E., Engelman A., Cherepanov P. (2009). A novel co-crystal structure affords the design of gain-of-function lentiviral integrase mutants in the presence of modified PSIP1/LEDGF/p75. PLoS Pathog..

[110] Cherepanov P., Sun Z.Y., Rahman S., Maertens G., Wagner G., Engelman A. (2005). Solution structure of the HIV-1 integrase-binding domain in LEDGF/p75. Nat. Struct. Mol. Biol..

[111] Hare S., Di Nunzio F., Labeja A., Wang J., Engelman A., Cherepanov P. (2009). Structural basis for functional tetramerization of lentiviral integrase. PLoS Pathog..

[112] Aiyer S., Swapna G.V.T., Malani N., Aramini J.M., Schneider W.M., Plumb M.R., Ghanem M., Larue R.C., Sharma A., Studamire B. (2014). Altering murine leukemia virus integration through disruption of the integrase and BET protein family interaction. Nucleic Acids Res..

[113] Roth M.J. (1991). Mutational analysis of the carboxyl terminus of the Moloney murine leukemia virus integration protein. J. Virol..

[114] Xie W., Gai X., Zhu Y., Zappulla D.C., Sternglanz R., Voytas D.F. (2001). Targeting of the yeast Ty5 retrotransposon to silent chromatin is mediated by interactions between integrase and Sir4p. Mol. Cell. Biol..

[115] Zhu Y., Dai J., Fuerst P.G., Voytas D.F. (2003). Controlling integration specificity of a yeast retrotransposon. Proc. Natl. Acad. Sci. U.S.A..

[116] Kessl J.J., Jena N., Koh Y., Taskent-Sezgin H., Slaughter A., Feng L., de Silva S., Wu L., Le Grice S.F., Engelman A. (2012). A multimode, cooperative mechanism of action of allosteric HIV-1 integrase inhibitors. J. Biol. Chem..

[117] Christ F., Shaw S., Demeulemeester J., Desimmie B.A., Marchand A., Butler S., Smets W., Chaltin P., Westby M., Debyser Z. (2012). Small-molecule inhibitors of the LEDGF/p75 binding site of integrase block HIV replication and modulate integrase multimerization. Antimicrob. Agents Chemother..

[118] Tsiang M., Jones G.S., Niedziela-Majka A., Kan E., Lansdon E.B., Huang W., Hung M., Samuel D., Novikov N., Xu Y. (2012). New class of HIV-1 integrase (IN) inhibitors with a dual mode of action. J. Biol. Chem..

[119] Pradeepa M.M., Sutherland H.G., Ule J., Grimes G.R., Bickmore W.A. (2012). Psip1/Ledgf p52 binds methylated histone H3K36 and splicing factors and contributes to the regulation of alternative splicing. PLoS Genet..

[120] Eidahl J.O., Crowe B.L., North J.A., McKee C.J., Shkriabai N., Feng L., Plumb M., Graham R.L., Gorelick R.J., Hess S. (2013). Structural basis for high-affinity binding of LEDGF PWWP to mononucleosomes. Nucleic Acids Res..

[121] van Nuland R., van Schaik F.M., Simonis M., van Heesch S., Cuppen E., Boelens R., Timmers H.M., van Ingen H. (2013). Nucleosomal DNA binding drives the recognition of H3K36-methylated nucleosomes by the PSIP1-PWWP domain. Epigenetics Chromatin.

[122] Roth S.L., Malani N., Bushman F.D. (2011). Gammaretroviral integration into nucleosomal target DNA in vivo. J. Virol..

[123] Dey A., Chitsaz F., Abbasi A., Misteli T., Ozato K. (2003). The double bromodomain protein Brd4 binds to acetylated chromatin during interphase and mitosis. Proc. Natl. Acad. Sci. U.S.A..

[124] Vollmuth F., Blankenfeldt W., Geyer M. (2009). Structures of the dual bromodomains of the P-TEFb-activating protein Brd4 at atomic resolution. J. Biol. Chem..

[125] Umehara T., Nakamura Y., Jang M.K., Nakano K., Tanaka A., Ozato K., Padmanabhan B., Yokoyama S. (2010). Structural basis for acetylated histone H4 recognition by the human BRD2 bromodomain. J. Biol. Chem..

[126] Zhang W., Prakash C., Sum C., Gong Y., Li Y., Kwok J.J.T., Thiessen N., Pettersson S., Jones S.J.M., Knapp S. (2012). Bromodomain-containing protein 4 (BRD4) regulates RNA polymerase II serine 2 phosphorylation in human CD4+ T cells. J. Biol. Chem..

[127] Gijsbers R., Vets S., De Rijck J., Ocwieja K.E., Ronen K., Malani N., Bushman F.D., Debyser Z. (2011). Role of the PWWP domain of lens epithelium-derived growth factor (LEDGF)/p75 cofactor in lentiviral integration targeting. J. Biol. Chem..

[128] Shun M.C., Botbol Y., Li X., Di Nunzio F., Daigle J.E., Yan N., Lieberman J., Lavigne M., Engelman A. (2008). Identification and characterization of PWWP domain residues critical for LEDGF/p75 chromatin binding and human immunodeficiency virus type 1 infectivity. J. Virol..

[129] Wang G.P., Ciuffi A., Leipzig J., Berry C.C., Bushman F.D. (2007). HIV integration site selection: analysis by massively parallel pyrosequencing reveals association with epigenetic modifications. Genome Res..

[130] Filippakopoulos P., Picaud S., Mangos M., Keates T., Lambert J.P., Barsyte-Lovejoy D., Felletar I., Volkmer R., Muller S., Pawson T. (2012). Histone recognition and large-scale structural analysis of the human bromodomain family. Cell.

[131] Calo E., Wysocka J. (2013). Modification of enhancer chromatin: what, how, and why. Mol. Cell.

[132] Ernst J., Kheradpour P., Mikkelsen T.S., Shoresh N., Ward L.D., Epstein C.B., Zhang X., Wang L., Issner R., Coyne M. (2011). Mapping and analysis of chromatin state dynamics in nine human cell types. Nature.

[133] Delmore J.E., Issa G.C., Lemieux M.E., Rahl P.B., Shi J., Jacobs H.M., Kastritis E., Gilpatrick T., Paranal R.M., Qi J. (2011). BET bromodomain inhibition as a therapeutic strategy to target c-Myc. Cell.

[134] Lovén J., Hoke H.A., Lin C.Y., Lau A., Orlando D.A., Vakoc C.R., Bradner J.E., Lee T.I., Young R.A. (2013). Selective inhibition of tumor oncogenes by disruption of super-enhancers. Cell.

[135] Anders L., Guenther M.G., Qi J., Fan Z.P., Marineau J.J., Rahl P.B., Lovén J., Sigova A.A., Smith W.B., Lee T.I. (2014). Genome-wide localization of small molecules. Nat. Biotechnol..

[136] Nakajima N., Lu R., Engelman A. (2001). Human immunodeficiency virus type 1 replication in the absence of integrase-mediated DNA recombination: definition of permissive and nonpermissive T-cell lines. J. Virol..

[137] Matreyek K.A., Engelman A. (2013). Viral and cellular requirements for the nuclear entry of retroviral preintegration nucleoprotein complexes. Viruses.

[138] Roe T., Reynolds T.C., Yu G., Brown P.O. (1993). Integration of murine leukemia virus DNA depends on mitosis. EMBO J..

[139] Yamashita M., Emerman M. (2004). Capsid is a dominant determinant of retrovirus infectivity in nondividing cells. J. Virol..

[140] Brass A.L., Dykxhoorn D.M., Benita Y., Yan N., Engelman A., Xavier R.J., Lieberman J., Elledge S.J. (2008). Identification of host proteins required for HIV infection through a functional genomic screen. Science.

[141] Konig R., Zhou Y., Elleder D., Diamond T.L., Bonamy G.M., Irelan J.T., Chiang C.Y., Tu B.P., De Jesus P.D., Lilley C.E. (2008). Global analysis of host-pathogen interactions that regulate early-stage HIV-1 replication. Cell.

[142] Zhou H., Xu M., Huang Q., Gates A.T., Zhang X.D., Castle J.C., Stec E., Ferrer M., Strulovici B., Hazuda D.J. (2008). Genome-scale RNAi screen for host factors required for HIV replication. Cell Host Microbe.

[143] Christ F., Thys W., De Rijck J., Gijsbers R., Albanese A., Arosio D., Emiliani S., Rain J.C., Benarous R., Cereseto A. (2008). Transportin-SR2 imports HIV into the nucleus. Curr. Biol..

[144] Ocwieja K.E., Brady T.L., Ronen K., Huegel A., Roth S.L., Schaller T., James L.C., Towers G.J., Young J.A., Chanda S.K. (2011). HIV integration targeting: a pathway involving Transportin-3 and the nuclear pore protein RanBP2. PLoS Pathog..

[145] Matreyek K.A., Yucel S.S., Li X., Engelman A. (2013). Nucleoporin NUP153 phenylalanine-glycine motifs engage a common binding pocket within the HIV-1 capsid protein to mediate lentiviral infectivity. PLoS Pathog..

[146] Koh Y., Wu X., Ferris A.L., Matreyek K.A., Smith S.J., Lee K., KewalRamani V.N., Hughes S.H., Engelman A. (2013). Differential effects of human immunodeficiency virus type 1 capsid and cellular factors nucleoporin 153 and LEDGF/p75 on the efficiency and specificity of viral DNA integration. J. Virol..

[147] Di Nunzio F., Fricke T., Miccio A., Valle-Casuso J.C., Perez P., Souque P., Rizzi E., Severgnini M., Mavilio F., Charneau P. (2013). Nup153 and Nup98 bind the HIV-1 core and contribute to the early steps of HIV-1 replication. Virology.

[148] Meehan A.M., Saenz D.T., Guevera R., Morrison J.H., Peretz M., Fadel H.J., Hamada M., van Deursen J., Poeschla E.M. (2014). A cyclophilin homology domain-independent role for Nup358 in HIV-1 infection. PLoS Pathog..

[149] Schaller T., Ocwieja K.E., Rasaiyaah J., Price A.J., Brady T.L., Roth S.L., Hue S., Fletcher A.J., Lee K., KewalRamani V.N. (2011). HIV-1 capsid-cyclophilin interactions determine nuclear import pathway, integration targeting and replication efficiency. PLoS Pathog..

[150] Di Primio C., Quercioli V., Allouch A., Gijsbers R., Christ F., Debyser Z., Arosio D., Cereseto A. (2013). Single-cell imaging of HIV-1 provirus (SCIP). Proc. Natl. Acad. Sci. U.S.A..

[151] Elis E., Ehrlich M., Prizan-Ravid A., Laham-Karam N., Bacharach E. (2012). p12 tethers the murine leukemia virus pre-integration complex to mitotic chromosomes. PLoS Pathog..

[152] Schneider W.M., Brzezinski J.D., Aiyer S., Malani N., Gyuricza M., Bushman F.D., Roth M.J. (2013). Viral DNA tethering domains complement replication-defective mutations in the p12 protein of MuLV Gag. Proc. Natl. Acad. Sci. U.S.A..

[153] Verma I.M., Weitzman M.D. (2005). Gene therapy: twenty-first century medicine. Annu. Rev. Biochem..

[154] Boztug K., Dewey R.A., Klein C. (2006). Development of hematopoietic stem cell gene therapy for Wiskott-Aldrich syndrome. Curr. Opin. Mol. Ther..

[155] Fischer A., Hacein-Bey-Abina S., Cavazzana-Calvo M. (2011). Gene therapy for primary adaptive immune deficiencies. J. Allergy Clin. Immunol..

[156] Rivat C., Santilli G., Gaspar H.B., Thrasher A.J. (2012). Gene therapy for primary immunodeficiencies. Hum. Gene Ther..

[157] Biasco L., Baricordi C., Aiuti A. (2012). Retroviral integrations in gene therapy trials. Mol. Ther..

[158] Wu C., Dunbar C.E. (2011). Stem cell gene therapy: the risks of insertional mutagenesis and approaches to minimize genotoxicity. Front. Med..

[159] Ott M.G., Schmidt M., Schwarzwaelder K., Stein S., Siler U., Koehl U., Glimm H., Kuhlcke K., Schilz A., Kunkel H. (2006). Correction of X-linked chronic granulomatous disease by gene therapy, augmented by insertional activation of MDS1-EVI1, PRDM16 or SETBP1. Nat. Med..

[160] Boztug K., Schmidt M., Schwarzer A., Banerjee P.P., Diez I.A., Dewey R.A., Bohm M., Nowrouzi A., Ball C.R., Glimm H. (2010). Stem-cell gene therapy for the Wiskott-Aldrich syndrome. N. Engl. J. Med..

[161] Stein S., Ott M.G., Schultze-Strasser S., Jauch A., Burwinkel B., Kinner A., Schmidt M., Kramer A., Schwable J., Glimm H. (2010). Genomic instability and myelodysplasia with monosomy 7 consequent to EVI1 activation after gene therapy for chronic granulomatous disease. Nat. Med..

[162] Lim K.I. (2012). Retroviral integration profiles: their determinants and implications for gene therapy. BMB Rep..

[163] Nienhuis A.W., Dunbar C.E., Sorrentino B.P. (2006). Genotoxicity of retroviral integration in hematopoietic cells. Mol. Ther..

[164] Trobridge G.D. (2011). Genotoxicity of retroviral hematopoietic stem cell gene therapy. Expert. Opin. Biol. Ther..

[165] Nowrouzi A., Glimm H., von Kalle C., Schmidt M. (2011). Retroviral vectors: post entry events and genomic alterations. Viruses.

[166] Knudson A.G. (2002). Cancer genetics. Am. J. Med. Genet..

[167] Stephens P.J., Greenman C.D., Fu B., Yang F., Bignell G.R., Mudie L.J., Pleasance E.D., Lau K.W., Beare D., Stebbings L.A. (2011). Massive genomic rearrangement acquired in a single catastrophic event during cancer development. Cell.

[168] Yu S.F., von Ruden T., Kantoff P.W., Garber C., Seiberg M., Ruther U., Anderson W.F., Wagner E.F., Gilboa E. (1986). Self-inactivating retroviral vectors designed for transfer of whole genes into mammalian cells. Proc. Natl. Acad. Sci. U.S.A..

[169] Montini E., Cesana D., Schmidt M., Sanvito F., Bartholomae C.C., Ranzani M., Benedicenti F., Sergi L.S., Ambrosi A., Ponzoni M. (2009). The genotoxic potential of retroviral vectors is strongly modulated by vector design and integration site selection in a mouse model of HSC gene therapy. J. Clin. Invest..

[170] Cavazza A., Cocchiarella F., Bartholomae C., Schmidt M., Pincelli C., Larcher F., Mavilio F. (2013). Self-inactivating MLV vectors have a reduced genotoxic profile in human epidermal keratinocytes. Gene Ther..

[171] Mukherjee S., Thrasher A.J. (2013). Gene therapy for PIDs: progress, pitfalls and prospects. Gene.

[172] Zhao Y., Yang C.Y., Wang S. (2013). The making of I-BET762, a BET bromodomain inhibitor now in clinical development. J. Med. Chem..

[173] American Associationfor Cancer Research (2014). Epigenetic therapy beneficial in blood cancers. Cancer Discovery.

[174] Donzella G.A., Jonsson C.B., Roth M.J. (1996). Coordinated disintegration reactions mediated by Moloney murine leukemia virus integrase. J. Virol..

[175] Jonsson C.B., Donzella G.A., Gaucan E., Smith C.M., Roth M.J. (1996). Functional domains of Moloney murine leukemia virus integrase defined by mutation and complementation analysis. J. Virol..

[176] Schneider W.M., Wu D.T., Amin V., Aiyer S., Roth M.J. (2012). MuLV IN mutants responsive to HDAC inhibitors enhance transcription from unintegrated retroviral DNA. Virology.

[177] Seamon J.A., Adams M., Sengupta S., Roth M.J. (2000). Differential effects of C-terminal molecular tagged integrase on replication competent moloney murine leukemia virus. Virology.

[178] Seamon J.A., Jones K.S., Miller C., Roth M.J. (2002). Inserting a nuclear targeting signal into a replication-competent Moloney murine leukemia virus affects viral export and is not sufficient for cell cycle-independent infection. J. Virol..

[179] Puglia J., Wang T., Smith-Snyder C., Cote M., Scher M., Pelletier J.N., John S., Jonsson C.B., Roth M.J. (2006). Revealing domain structure through linker-scanning analysis of the murine leukemia virus (MuLV) RNase H and MuLV and human immunodeficiency virus type 1 integrase proteins. J. Virol..

[180] Sakuma T., Barry M.A., Ikeda Y. (2012). Lentiviral vectors: basic to translational. Biochem J..

[181] Cavazzana-Calvo M., Payen E., Negre O., Wang G., Hehir K., Fusil F., Down J., Denaro M., Brady T., Westerman K. (2010). Transfusion independence and HMGA2 activation after gene therapy of human beta-thalassaemia. Nature.

[182] Wang H., Shun M.C., Li X., Di Nunzio F., Hare S., Cherepanov P., Engelman A. (2014). Efficient transduction of LEDGF/p75 mutant cells by complementary gain-of-function HIV-1 integrase mutant viruses. Mol.Ther. Methods Clin. Dev..

[183] Floyd S.R., Pacold M.E., Huang Q., Clarke S.M., Lam F.C., Cannell I.G., Bryson B.D., Rameseder J., Lee M.J., Blake E.J. (2013). The bromodomain protein Brd4 insulates chromatin from DNA damage signalling. Nature.

[184] Jenkins T.M., Esposito D., Engelman A., Craigie R. (1997). Critical contacts between HIV-1 integrase and viral DNA identified by structure-based analysis and photo-crosslinking. EMBO J..

[185] Schwartz R.M., Dayhoff M.O., Dayhoff MO (1978). Atlas of Protein Sequence and Structure.

[186] Metais J., Topp S., Doty R., Borate B., Nguyen A., Wolfsberg T., Abkowitz J., Dunbar C. (2010). Feline leukemia virus integrase and capsid packaging functions do not change the insertion profile of standard Moloney retroviral vectors. Gene Ther..

